# Heparin Protects Human Neural Progenitor Cells from Zika Virus-Induced Cell Death While Preserving Their Differentiation into Mature Neuroglial Cells

**DOI:** 10.1128/jvi.01122-22

**Published:** 2022-09-19

**Authors:** Isabel Pagani, Linda Ottoboni, Paola Podini, Silvia Ghezzi, Elena Brambilla, Svetlana Bezukladova, Davide Corti, Marco Emilio Bianchi, Maria Rosaria Capobianchi, Guido Poli, Paola Panina-Bordignon, Edwin A. Yates, Gianvito Martino, Elisa Vicenzi

**Affiliations:** a San Raffaele Scientific Institute, Milan, Italy; b Humabs Biomed SA, a subsidiary of Vir Biotechnology, Bellinzona, Switzerland; c Vita-Salute San Raffaele University, School of Medicine, Milan, Italy; d National Institute for Infectious Diseases, “Lazzaro Spallanzani,” IRCCS, Rome, Italy; e Department of Biochemistry and Systems Biology, ISMIB, University of Liverpool, Liverpool, United Kingdom; University of North Carolina at Chapel Hill

**Keywords:** ZIKV, cell death, cell differentiation, heparin, neural progenitor cells, neuroglial cells

## Abstract

Zika virus (ZIKV) is an arbovirus member of the *Flaviviridae* family that causes severe congenital brain anomalies in infected fetuses. The key target cells of ZIKV infection, human neural progenitor cells (hNPCs), are highly permissive to infection that causes the inhibition of cell proliferation and induces cell death. We have previously shown that pharmaceutical-grade heparin inhibits virus-induced cell death with negligible effects on *in vitro* virus replication in ZIKV-infected hNPCs at the “high” multiplicity of infection (MOI) of 1. Here, we show that heparin inhibits formation of ZIKV-induced intracellular vacuoles, a signature of paraptosis, and inhibits necrosis and apoptosis of hNPCs grown as neurospheres (NS). To test whether heparin preserved the differentiation of ZIKV-infected hNPCs into neuroglial cells, hNPCs were infected at the MOI of 0.001. In this experimental condition, heparin inhibited ZIKV replication by ca. 2 log_10_, mostly interfering with virion attachment, while maintaining its protective effect against ZIKV-induced cytopathicity. Heparin preserved differentiation into neuroglial cells of hNPCs that were obtained from either human-induced pluripotent stem cells (hiPSC) or by fetal tissue. Quite surprisingly, multiple additions of heparin to hNPCs enabled prolonged virus replication while preventing virus-induced cytopathicity. Collectively, these results highlight the potential neuroprotective effect of heparin that could serve as a lead compound to develop novel agents for preventing the damage of ZIKV infection on the developing brain.

**IMPORTANCE** ZIKV is a neurotropic virus that invades neural progenitor cells (NPCs), causing inhibition of their proliferation and maturation into neurons and glial cells. We have shown previously that heparin, an anticoagulant also used widely during pregnancy, prevents ZIKV-induced cell death with negligible inhibition of virus replication. Here, we demonstrate that heparin also exerts antiviral activity against ZIKV replication using a much lower infectious inoculum. Moreover, heparin interferes with different modalities of virus-induced cell death. Finally, heparin-induced prevention of virus-induced NPC death allows their differentiation into neuroglial cells despite the intracellular accumulation of virions. These results highlight the potential use of heparin, or pharmacological agents derived from it, in pregnant women to prevent the devastating effects of ZIKV infection on the developing brain of their fetuses.

## INTRODUCTION

Zika virus (ZIKV) is a member of the *Flaviviridae* family that is mainly transmitted to humans through the mosquito bites of the *Aedes* species (1), but it can also spread through sexual transmission (2, 3), the maternal-fetal interface, and blood transfusion (4). Following its discovery in 1947 (5), infections of humans by ZIKV were reported occasionally in Africa and Asia, typically with mild clinical presentations (6). After 70 years of limited ZIKV spread with a few outbreaks, such as those in the Pacific Islands in 2007 (7) and French Polynesia in 2013 (8), a significant challenge emerged to global health authorities in February 2016 following the unexpected outbreak of neonates presenting abnormally small brains (microcephaly) in Brazil (9, 10). The widespread infection of pregnant women with ZIKV had caused serious birth defects, including neurological diseases (11–13). *In utero* ZIKV-associated pathological conditions of the fetus were supported by the evidence of ZIKV particles visible in brain sections using transmission electron microscopy and recovery of the full ZIKV genome from the fetal brain (14). ZIKV was detected in neural and nonneural cells by immunolabelling of fetal brain from aborted fetuses, although the highest rate of infection was in intermediate progenitor cells and immature neurons (15), suggesting a strong viral tropism for human neural progenitor cells (hNPCs). Numerous reports have demonstrated that hNPCs derived from human induced pluripotent stem cells (hiPSCs) are highly permissive to ZIKV infection *in vitro* causing inhibition of their proliferation and cell death (16–23). These findings were also reproduced in mouse animal models that further demonstrated the development of microcephaly in infected fetuses (24–26).

Several studies have focused on the modalities of ZIKV-induced cell death, and both apoptotic and nonapoptotic modalities have been described as mechanisms that lead hNPCs to die consequent to virus replication (27, 28). In this regard, ZIKV, like other flaviviruses, exploits the endoplasmic reticulum (ER) to assemble the replication complex with an expansion of cellular membranes that contain newly formed immature virions (29). This overwhelming ER load leads to ER stress with consequent stimulation of the unfolded protein response (UPR) (30) and, in particular, expression of the C/EBP homologous protein (CHOP) that initiates apoptosis in infected cells (31). Prolonged ER stress upon ZIKV infection, however, also triggers a particular type of nonapoptotic cell death characterized by the cytoplasmic formation of ER-derived vacuoles leading to paraptosis-like cell death (32); paraptosis was firstly defined by morphological criteria and by occurring independently of the activation of cleaved-caspase 3 (cl-CASP3) (33). A hallmark of nonapoptotic cell death is the expression of high mobility group 1 (HMGB1) protein (34, 35), originally described as a DNA binding protein (36) involved in multiple nuclear functions, such as transcription, replication, recombination, DNA repair, and genomic stability (37). However, upon necrotic cell death, HMGB1 can be passively released into the extracellular environment where it acts as a damage-associated molecular pattern (DAMP) molecule triggering or potentiating the inflammatory response (38).

Following the strategy of repurposing drugs already approved for other medical indications, we have previously described the ability of heparin to inhibit ZIKV cytopathic effects without affecting virus replication in hNPCs when the infection was performed at the “high” multiplicity of infection (MOI) of 1 (39). In the present study, we have tested whether heparin protects from virus-induced apoptotic and nonapoptotic/paraptosis-like cell death in infected stem-cell-derived neural progenitors and its antiviral activity when cells were infected with a significantly lower MOI (0.001). As hiPSC-derived NPCs do not consistently recapitulate *in vivo* embryonic human brain gene expression (40), we have also investigated ZIKV infection in hNPCs obtained directly from human fetal (hf) tissue (15, 22). As both hiPSC-NPCs and hf-NPCs spontaneously aggregate to form neurospheres (NS) in nonadherent culture conditions, they have been used as a surrogate model of neurogenesis and as targets of ZIKV infection (17, 21). Thus, we have exploited NS from both hiPSC-NPCs and hf-NPCs to determine the effect of heparin protection from ZIKV infection and its capacity to permit hNPC differentiation into mature neuroglial cells. Heparin was tested prior to, or following, infection and was repeatedly added during the differentiation experiments.

Our results highlight the potential of heparin not only to interfere with ZIKV infection and replication (at low MOI) but also to protect hNPCs against the cytopathic effects of ZIKV, allowing their differentiation into neuroglial cells.

## RESULTS

### Heparin inhibits ZIKV-induced nonapoptotic cell death.

**FIG 1:**
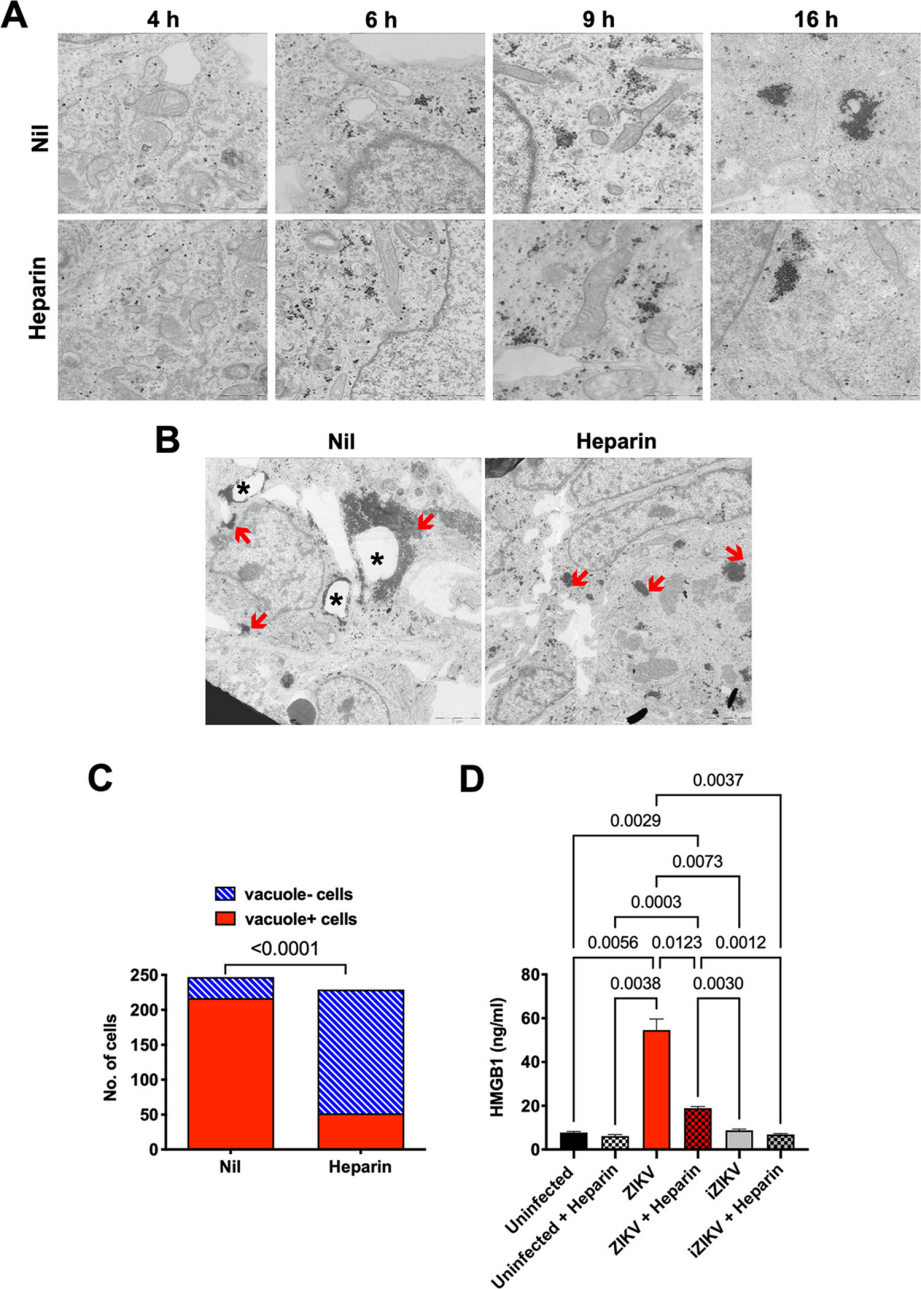
Heparin prevents the formation of cytoplasmic vacuoles in ZIKV-infected hiPSC-derived NPCs. (A) Electron microscopy specimens of hiPSC-NPCs treated, or not, with heparin (100 μg/mL) 1 h prior to infection with the PRVABC59 isolate (MOI of 1). Infected hiPSC-NPCs were fixed at 4, 6, 9, and 16 h postinfection. Increased accumulation of clusters of ZIKV particles was visible in untreated and treated infected hiPSC-NPCs; bar = 2 μm. (B) Electron microscopy specimens of hiPSC-NPCs treated, or not, with heparin (100 μg/mL) 3 days postinfection. Asterisks indicate vacuoles, and clusters of ZIKV particles are indicated with red arrows; bar = 2 μm. (C) Quantification of vacuoles in hiPSC-NPCs treated, or not, with heparin prior to infection with the PRVABC59 isolate. Bars represent vacuole-positive (red) and vacuole-negative (blue) cells counted in more than 200 images taken from 3 independent experiments. *P* value was calculated by Fisher’s exact test. (D) Levels of HMGB1 released in the culture supernatant after 6 days in uninfected condition, ZIKV infection, and exposure to heat-inactivated ZIKV (iZIKV). Bars represent the mean ± standard deviation (SD) of 3 independent experiments. *P* values were calculated by one-way ANOVA with the Bonferroni correction.

We have previously demonstrated that heparin prevents ZIKV-induced cell death in hNPCs grown as monolayers (39). Since a recent report highlighted a mechanism of massive cytoplasmic vacuolization that leads to paraptosis-like death in ZIKV-infected HeLa cells, human foreskin fibroblasts, and astrocytes (32), we tested whether these phenomena could also be observed upon ZIKV infection of hiPSC-NPCs and whether heparin could affect virus-induced vacuole formation. Firstly, cells infected with the PRVABC59 isolate were examined at different time points postinfection by transmission electron microscopy. Single virions were visible in the cytoplasm and in the lysosomes shortly after infection (4 h), aggregated, and then localized around membrane-delimited vacuoles at 9 and 16 h postinfection. Occasionally, virions were contained inside the vacuoles, but the majority were located outside (Fig. 1A). Incubation of hiPSC-NPCs with heparin 1 h prior to infection did not alter the virion density or their subcellular localization compared to untreated infected cells until 16 h postinfection (Fig. 1A). The number of vacuoles increased at 72 h postinfection in the infected control cell cultures, whereas heparin reduced virion-associated vacuoles, and cells exhibited healthy and normal-appearing mitochondria as in uninfected conditions (Fig. 1B). To verify whether vacuole formation was induced in hiPSC-NPCs, virion-positive cells were examined for the presence of vacuoles. A total of 217 out of 247 ZIKV-infected cells (88%) were indeed vacuole positive, whereas heparin significantly reduced the number of vacuole-positive cells to 52 out of 229 (23%) (Fig. 1C).

As membrane damage causes the passive release of HMGB1, a marker of nonapoptotic cell death (41), its presence was measured in the culture supernatant of both control uninfected and ZIKV-infected hiPSC-NPCs in the presence or absence of heparin. HMGB1 was present at low levels in the cell culture supernatant of uninfected hiPSC-NPCs, whereas ZIKV significantly increased its levels 6 days postinfection (Fig. 1D), while heparin significantly lowered the levels of released HMGB1 in infected cells. As an additional control, we tested whether heparin interfered with the release of HMGB1 potentially induced by nonreplicating heat-inactivated ZIKV (iZIKV). As shown in Fig. 1D, cells exposed to iZIKV did not release HMGB1 at levels higher than those of uninfected cells, and heparin did not modify this profile, suggesting that heparin inhibits HMGB1 release only in productively infected cells undergoing viral-induced cytopathicity.

Overall, these results indicate that heparin prevents or diminishes nonapoptotic cell death caused by ZIKV infection, including paraptosis-like cytopathicity.

### Heparin inhibits both necrosis and apoptosis in ZIKV-infected neurospheres of different cellular origin.

**FIG 2:**
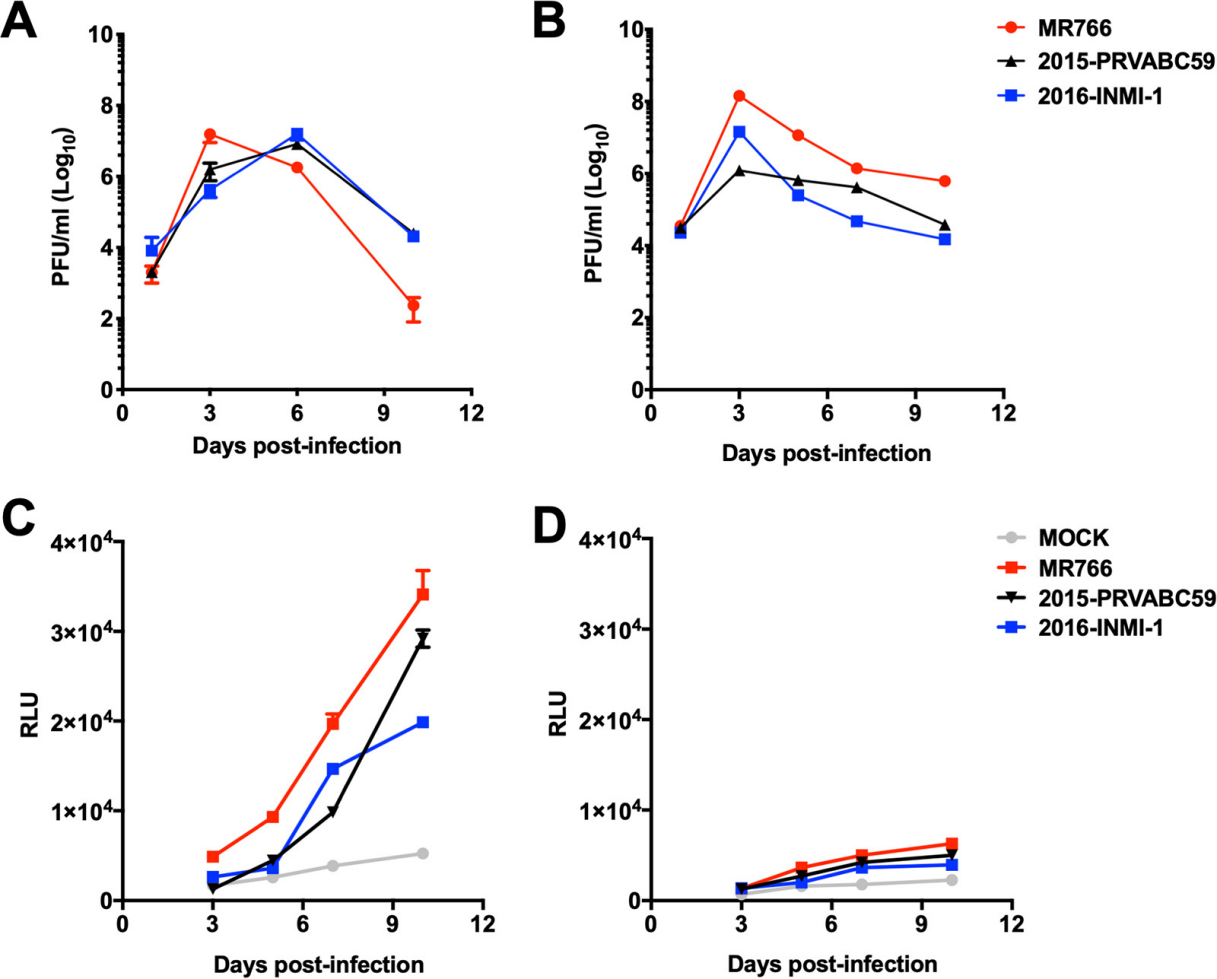
Kinetics of ZIKV replication and cytopathic effect in hiPSC-NPCs and hf-NPCs. hiPSC-NPCs (A) and hf-NPCs (B) were infected at the MOI of 1 with 3 different ZIKV isolates as follows: the historical MR766 and two recent strains, i.e., 2015-PRVABC59 and 2016-INMI-1 isolates. Supernatants were collected at 1, 3, 6, and 10 days postinfection. Infectious titers were determined by PFA in Vero cells. The AK activity was measured in supernatants of infected hiPSC-NPCs (C) and hf-NPCs (D). The results are expressed as relative luminescent units (RLU). Means ± SD of one experiment in triplicate is shown.

In order to validate heparin protection of ZIKV-induced cell damage observed in hiPSC-NPCs grown as a monolayer, a more complex three-dimensional (3D) system of NS formed by hNPC aggregates (42) was infected with ZIKV in the presence or absence of heparin. In addition to hiPSC-NS, NS were obtained from hNPCs isolated from a human fetal brain (hf-NS). Firstly, to determine the efficiency of ZIKV infection in hf-NPCs compared to hiPSC-NPCs, cells in monolayer were infected with three ZIKV isolates including the original African MR766 and the two more recent ones, i.e., Puerto Rico 2015-PRVABC59 and the Brazilian 2016-INMI-1. The peak of virus replication was reached between days 3 and 6 postinfection in both cell systems, with an increase of ca. 2 log_10_ of infectious virus released in the culture supernatant. In hiPSC-NPCs, a decrease of the viral titers was detected at day 10 postinfection (when the culture was terminated), being more prominent with the infection of MR766 than the two more recent isolates (Fig. 2A). However, in hf-NPCs, infectious virus persisted up to 10 days postinfection (Fig. 2B) with consistently lower levels of cytopathicity as measured by the adenylate kinase (AK) activity released in the supernatant of hf-NPCs compared to hiPSC-NPCs (Fig. 2D and C, respectively). The contemporary Brazilian 2016-INMI-1 isolate was selected for the next NS infection experiment.

**FIG 3:**
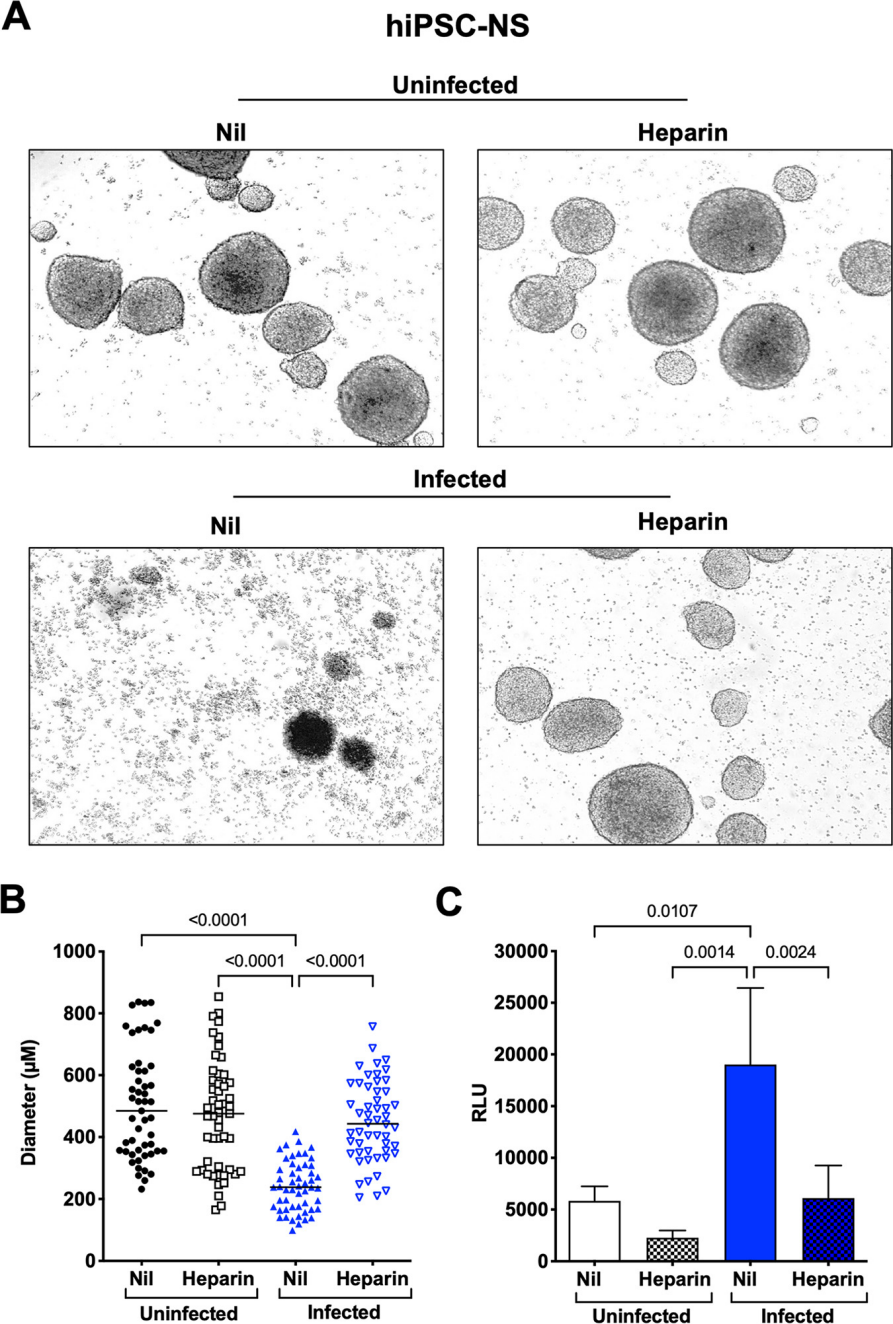
Heparin inhibits disruption of ZIKV-infected hiPSC-derived NS. (A) hiPSC-NS were treated with heparin (100 μg/mL) 1 h prior to infection with the Brazilian 2016-INMI-1 isolate (MOI of 1). After 4 h of ZIKV adsorption, virus inoculum was removed and fresh medium was replenished. Bright field photomicrographs of mock-treated versus heparin in both uninfected and infected conditions were taken 6 days postinfection. Calibration bars, 150 μm. (B) ZIKV caused a significant reduction in the NS number and size that was reverted by heparin to the levels of control cultures. Individual NS diameter values with the mean ± SD are shown. *P* values were calculated by one-way ANOVA with the Bonferroni correction. (C) Supernatants were collected 6 days postinfection and tested for AK activity. The results are expressed as relative luminescent units (RLU). Bars represent the mean ± SD of 5 independent experiments. *P* values were calculated by one-way ANOVA with the Bonferroni correction.

hiPSC-NS spontaneously formed from hNPCs after 3 days of culture in rotation without coating of extracellular matrix were incubated with heparin 1 h prior to infection with the Brazilian 2016-INMI-1 isolate. The number and diameter of hiPSC-NS were evaluated 3 days postinfection. ZIKV infection caused a marked reduction of hiPSC-NS number and size, whereas heparin reversed this effect almost to the levels of uninfected NS (Fig. 3A). Indeed, the diameter of uninfected hiPSC-NS had an average length of 513 ± 188 μm, which was not statistically different from that of heparin-treated uninfected NS, whereas ZIKV-infected NS had a significantly smaller average length (241 ± 80 μm). Interestingly, heparin preserved the diameter of the infected NS to the levels of uninfected ones (449 ± 130 μm) (Fig. 3B). To determine whether this size reduction was caused by virus-induced cell death, the extent of ZIKV-induced cell necrosis was determined. The reduction of number and size of infected NS was consistent with the levels of cell necrosis measured by the AK activity released in culture supernatant, which significantly increased in infected cultures, whereas heparin decreased the AK activity levels to those of the control cultures (Fig. 3C).

**FIG 4:**
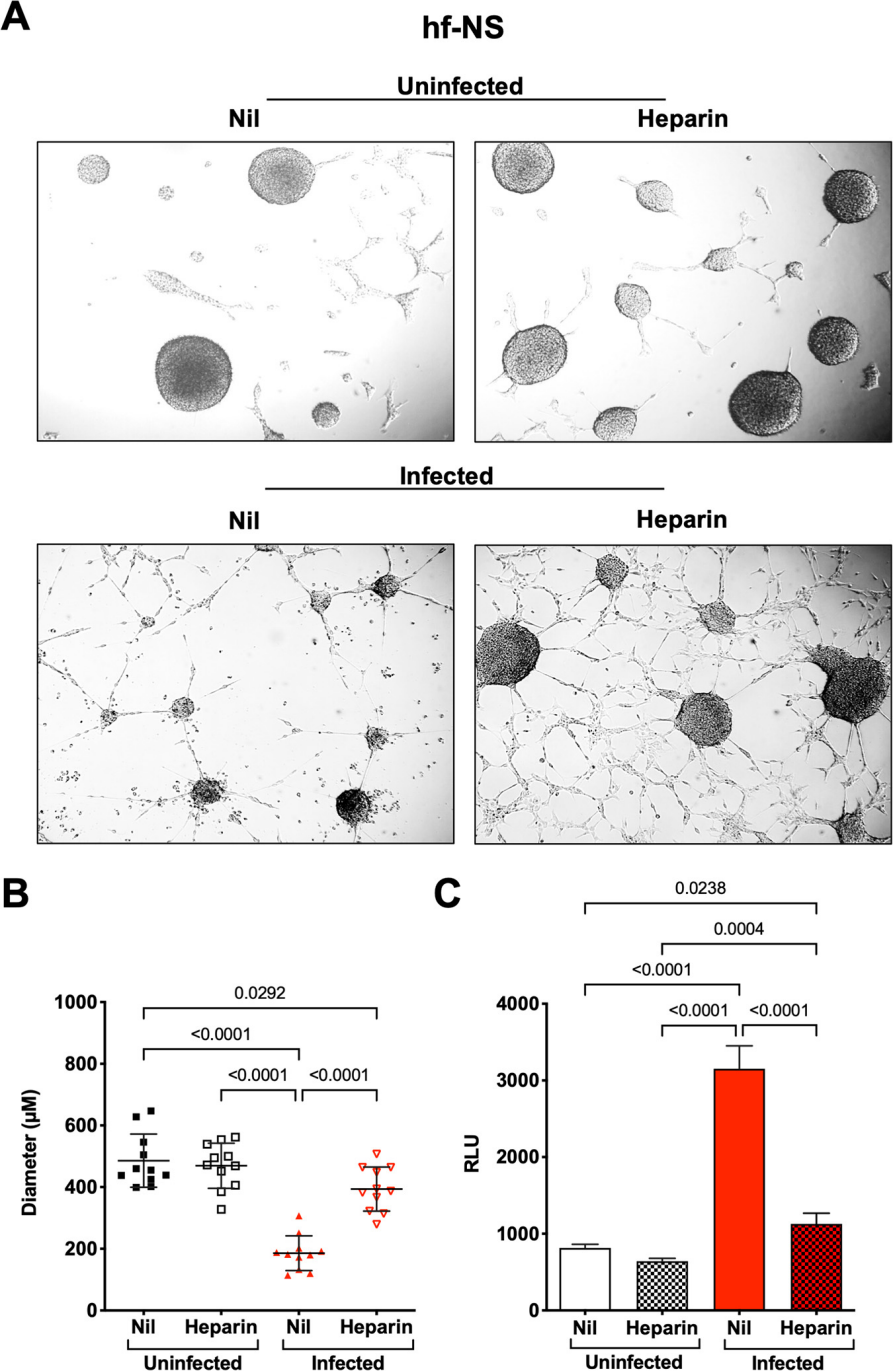
Heparin inhibits disruption of ZIKV-infected hf-NS. (A) hf-NS were treated with heparin (100 μg/mL) 1 h prior to infection with the Brazilian 2016-INMI-1 isolate (MOI of 1). After 4 h of ZIKV adsorption, virus inoculum was removed and the medium was replenished. Bright field photomicrographs of mock-treated versus heparin in both uninfected and infected conditions were taken 6 days postinfection. Calibration bars, 150 μm. (B) ZIKV caused a significant reduction in NS number and size that was reverted by heparin to the levels of control cultures. Individual NS diameter values with the mean ± SD are shown. *P* values were calculated by one-way ANOVA with the Bonferroni correction. (C) Supernatant of infected hf-NS with the Brazilian 2016-INMI-1 isolate was collected 6 days postinfection and tested for AK activity. The results are expressed as relative luminescent unit (RLU). Bars represent the mean ± SD of 3 independent experiments.

Similarly, ZIKV infection induced a reduction in number and size of hf-NS (Fig. 4A). Uninfected culture cells had an average diameter of 485 ± 86 μm, whereas that of infected hf-NS was significantly reduced (185 ± 56 μm). Heparin partially preserved the diameter of the hf-NS to that of the uninfected treated control (393 ± 71 μm) (Fig. 4B). As shown in Fig. 4C, the amount of AK activity released in the culture supernatant significantly increased in infected cultures, whereas heparin decreased the AK activity to that of control cultures.

**FIG 5:**
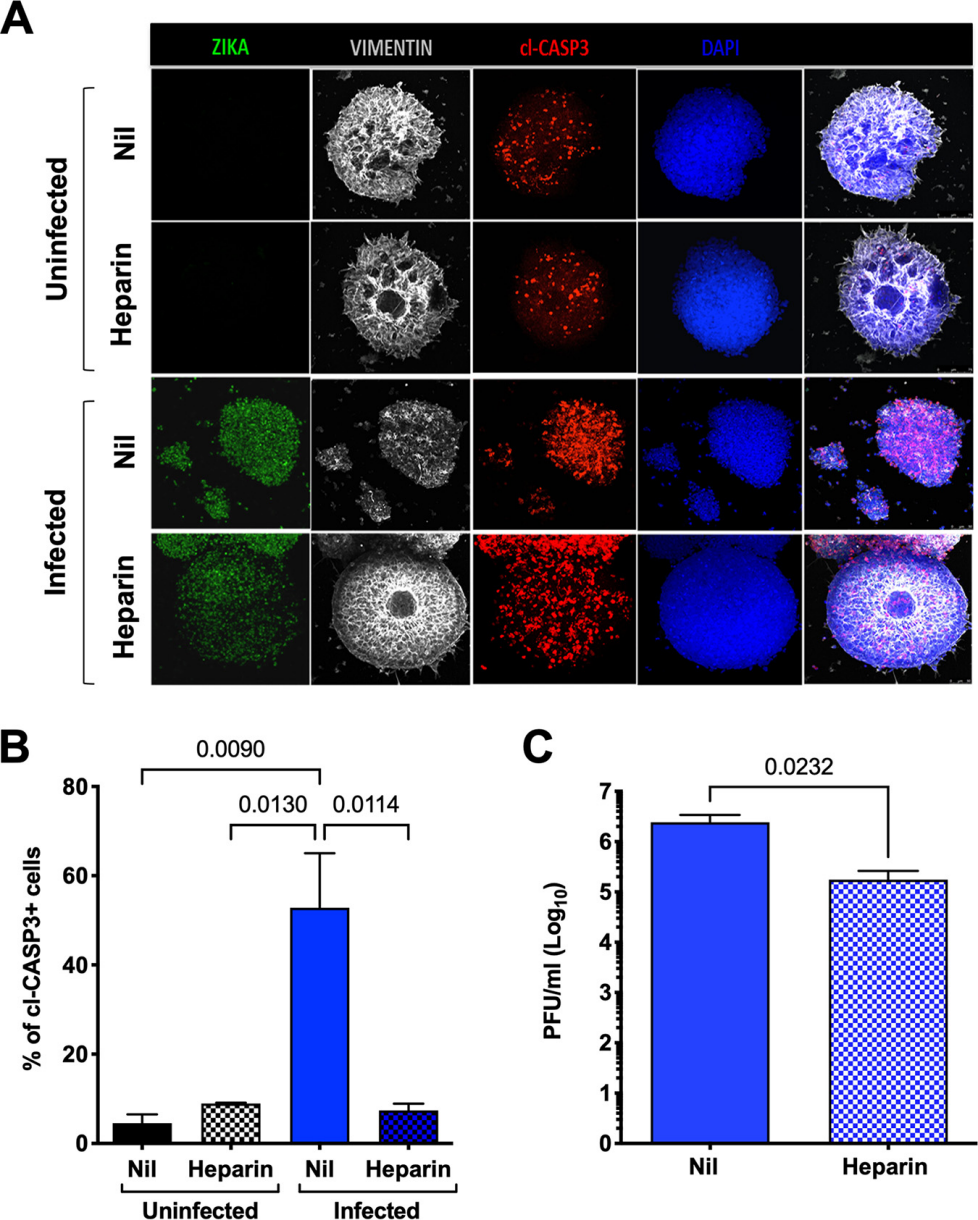
Heparin preserves hiPSC-NS structural integrity. (A) hiPSC-NS were treated with heparin 1 h prior to infection with the 2016-INMI-1 isolate (MOI of 1). After 4 h of ZIKV adsorption, virus inoculum was removed, and the medium was replenished. hiPSC-NS were fixed after 6 days postinfection. Immunostaining for dsRNA (green), vimentin (white), and cl-CASP3 (red) counterstained with DAPI (blue) in uninfected, ZIKV-infected, and heparin-treated NS. Scale bar, 50 μm. (B) hiPSC-NS were fixed after 4 and 6 days postinfection and immunostained for cl-CASP3 antibody. Scale bar, 50 μm. Images were obtained using an SP8 confocal microscope. Z-stack analyses were performed on 3 independent images for each condition and time points. (C) Six days postinfection, infectious viral titers were determined in the supernatant by PFA. Results are expressed as log_10_ of plaque forming unit/milliliter (PFU/mL). Bars represent the mean ± SD of 2 independent experiments in duplicate. *P* value was calculated by the Student's paired *t* test.

Since heparin treatment protected NS from ZIKV-induced disruption, the architecture of hiPSC-NS was determined by immunofluorescence. As shown in Fig. 5A, staining of vimentin, a type III intermediate filament of the cytoskeleton, formed a web inside uninfected NS. Notably, basal levels of apoptosis were observed in the core of uninfected NS, as described previously (43). Six days after infection, however, infected hiPSC-NS lost their integrity, and vimentin appeared dot-like resembling agglomeration. Conversely, when NS were incubated with heparin prior to infection, the vimentin web was still maintained even in infected cells.

To investigate whether ZIKV infection of NS could trigger apoptotic cell death, NS were stained with an anti-cl-CASP3 monoclonal antibody (MAb). Indeed, apoptosis was induced spontaneously in the core of uninfected NS as described previously (43), whereas ZIKV infection caused a significant increase of cl-CASP-3-positive cells (Fig. 5B). As for other forms of ZIKV-induced cell death, heparin also prevented apoptosis of NS (Fig. 5A and B). We also tested whether the amount of infectious virus released into the culture supernatant was modified in heparin-treated NS. Indeed, the infectious titers were significantly lowered by approximately 10-fold in heparin-treated NS compared to those in infected untreated NS (Fig. 5C), suggesting that heparin might also inhibit viral entry and replication as has been reported previously for other viral infections including severe acute respiratory syndrome coronavirus 2 (SARS-CoV-2) (44, 45).

Thus, heparin protects from ZIKV-induced necrotic and apoptotic cytopathicity of NS derived from different cellular origin and interferes with virus replication in these 3D models of NPC culture.

### Heparin inhibits early steps of ZIKV replication at low MOI.

**FIG 6:**
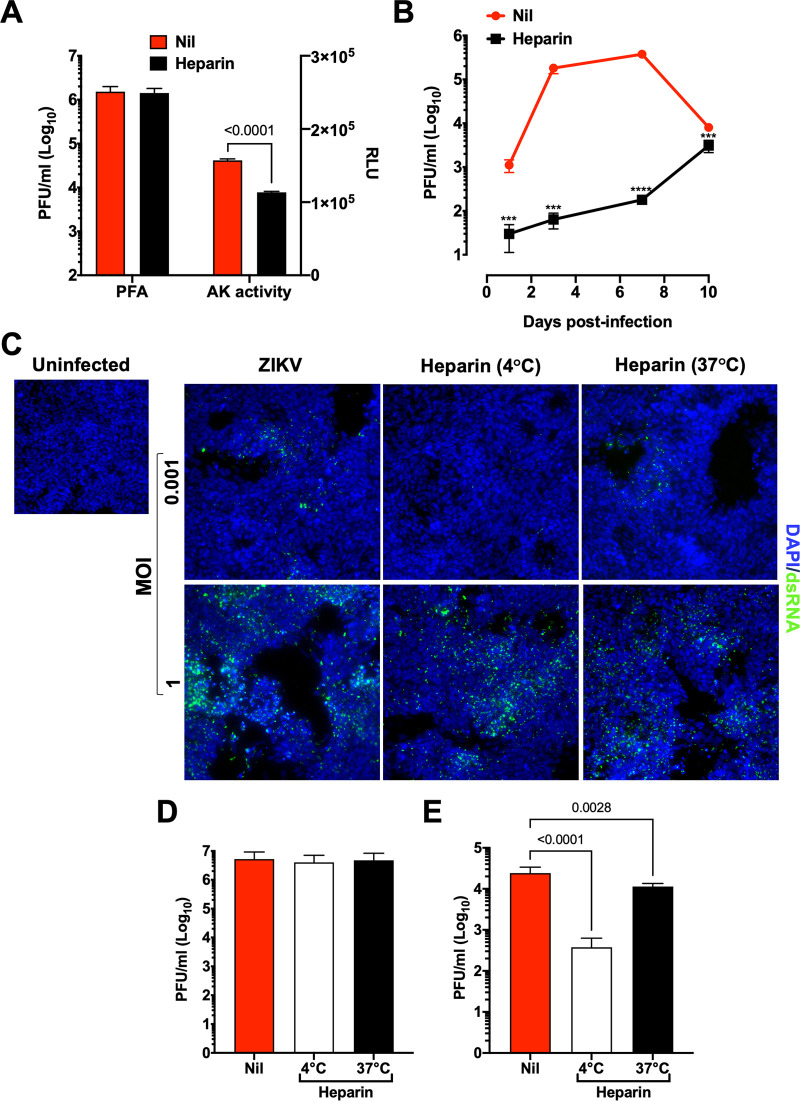
Heparin inhibits early steps of ZIKV entry. (A) hiPSC-derived NPCs were treated, or not, with heparin (100 μg/mL) 1 h prior to infection with the PRVABC59 isolate at the MOI of 1. Supernatants were collected 3 days postinfection and tested for both infectious viral titers and AK activity. Bars represent the mean ± SD of 3 independent experiments. (B) hiPSC-NPCs were treated, or not, with heparin (100 μg/mL) 1 h prior to infection with the PRVABC59 isolate at the MOI of 0.001. Kinetics of viral replication were determined by measuring the infectious virus released in the culture supernatant by PFA. A paired *t* test was used to determine the relationship between Nil and heparin treatment in each time point. ***, *P* value < 0.001; ****, *P* value < 0.0001. (C) To determine heparin inhibition of viral entry, hiPSC-NPCs were infected with PRVABC59 isolate at either an MOI of 1 or 0.001 for 3 h. Cells were treated with heparin (100 μg/mL) either before infection at 4°C (1 h; attachment step) or after infection at 37°C (15 min; entry step). Immunofluorescence analysis was performed 3 days postinfection. (D) Supernatants of hiPSC-NPCs infection at the MOI of 1 were collected at 3 days postinfection and tested by PFA. (E) Supernatants of hiPSC-NPCs infection at the MOI of 0.001 were collected at 3 days postinfection and tested by PFA. Bars represent the mean ± SD of 2 independent experiments in triplicate. *P* values were calculated by one-way ANOVA with the Bonferroni correction.

To test whether heparin could exert antiviral effects also in hNPCs grown in adherent conditions, hNPCs were infected with ZIKV after incubation with a “high” and a “low” MOI of 1 and 0.001, respectively. In agreement with our previous study, heparin did not inhibit ZIKV replication at the MOI of 1, although it inhibited ZIKV-induced cytopathic effects as measured by the levels of the AK activity in the culture supernatant (Fig. 6A). Conversely, a decrease of ca. 2 log_10_ of infectious virus was observed in heparin-treated cultures at the MOI of 0.001 (Fig. 6B).

As heparin has been previously reported to inhibit the early steps of viral infection, including the attachment/binding to specific receptors on target cells (44, 45), hNPCs were precooled and incubated with the virus at 4°C. Under these conditions, the virus attaches to target cells and binds to specific receptors, but the temperature-dependent step of viral entry is inhibited (46). Thus, to test whether heparin prevented ZIKV attachment/binding to target cells, heparin was added 1 h prior to the viral inoculum and maintained at 4°C together with the virus. Then, the cell cultures were incubated at 37°C to allow viral entry. Conversely, heparin was added to ZIKV-exposed cells only during their incubation at 37°C. hiPSC-NPCs were infected with MOIs of either 1 or 0.001, and virus replication was measured by immunofluorescence and plaque forming assay (PFA) 3 days after infection. A reduction of ZIKV-positive cells was observed in the presence of heparin when cells were infected with the low MOI, especially during the attachment phase (4°C); conversely, with the MOI of 1, the number of positive cells at 4°C was similar to that at 37°C, although the monolayer was less damaged in the presence of heparin (Fig. 6C). At the MOI of 1, heparin did not reduce the titer of released infectious virus when added at either 4°C or 37°C (Fig. 6D), whereas at the low MOI, heparin inhibited the virus titers by 2 log_10_ when it was added at 4°C, and an inhibitory effect on virus replication was also observed when cells were incubated at 37°C (Fig. 6E).

Taken together, these results demonstrate that heparin has antiviral effects in hNPCs grown in adherent conditions if the infectious titer of the inoculum is “low,” whereas at high MOI, it was cytoprotective in the absence of antiviral effects as originally described (39).

### Heparin preserves the capacity of hiPSC-NPCs to differentiate into neural cells when added either prior to, or after, ZIKV infection.

**FIG 7:**
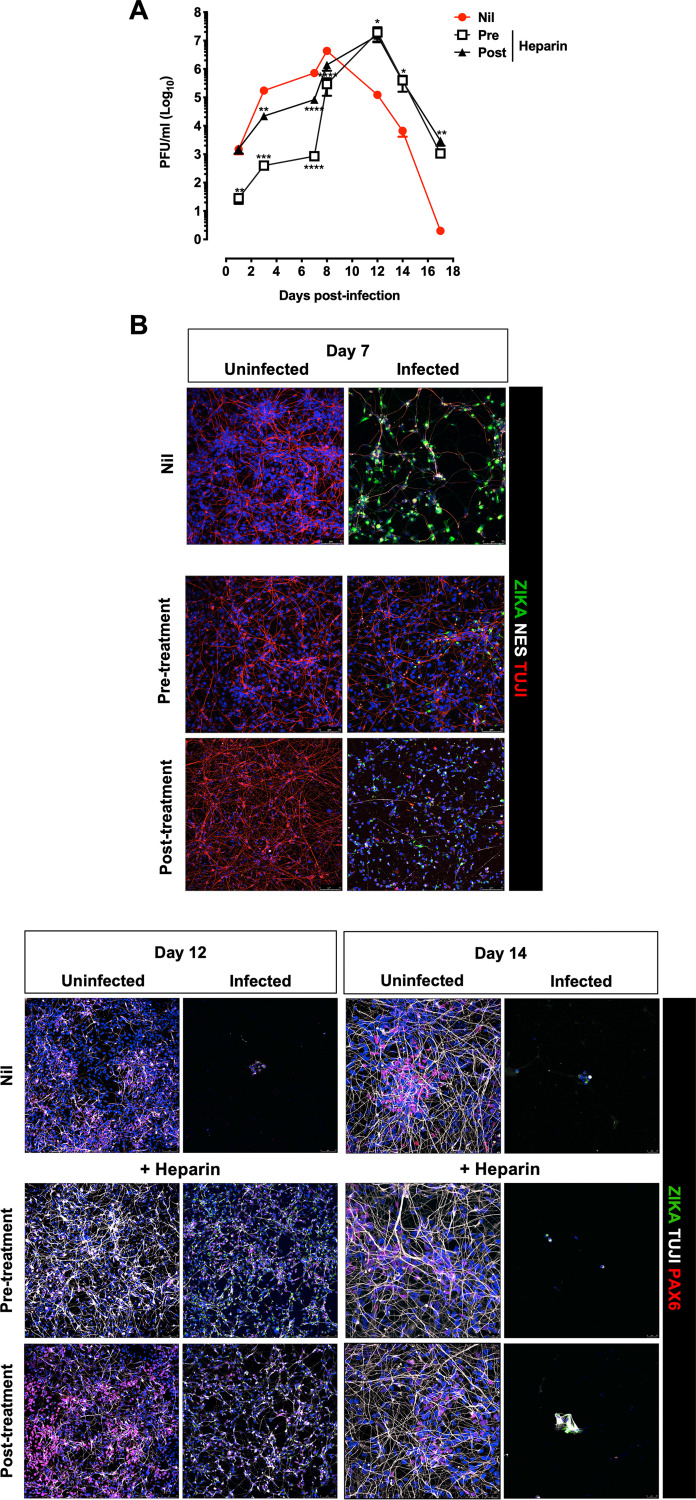
Kinetics of ZIKV replication in hiPSC-NPCs treated with a single dose of heparin. (A) hiPSC-derived NPCs were treated with heparin (100 μg/mL) before and after infection (1 h) with the PRVABC59 isolate (MOI, 0.001). Cell cultures were maintained in induction medium for 1 week and then in differentiation medium for at least 14 days. Kinetics of viral replication were determined by titering the infectious virus released in culture supernatants by PFA. The graph represents the mean ± SD of 3 independent experiments. One-way ANOVA was used with Bonferroni correction. *, Statistical comparison among groups (*, *P* < 0.05; **, *P* < 0.01; ***, *P* < 0.001; ****, *P* < 0.0001). (B) Differentiated hiPSC-NPCs were fixed at days 7, 12, and 14 postinfection and stained for ZIKV with envelope protein (ZIKA-green); neurons with beta tubulin III (TUJ1-white) and PAX6 (red). Blue channel is DAPI staining for all cell types. Scale bar, 75 μm and 25 μm. Images were obtained using SP8 confocal microscope.

Next, we tested whether heparin preserved the capacity of infected hiPSC-NPC to differentiate into mature neuroglial cells (47–49). To this end, differentiation of hiPSC-NPCs was induced following a two-step approach modified from Muratore et al. (50) beginning 4 h after incubation with ZIKV. The low MOI of 0.001 was selected to prolong cell viability in untreated conditions to at least 21 days of culture. After removal of the virus inoculum, hiPSC-NPCs were maintained in induction medium for 7 days, followed by a 14-day culture in differentiation medium (50). Cells were incubated with heparin either 1 h prior to infection (pretreatment) or 4 h after removal of the virus inoculum (posttreatment). Indirect immunofluorescence was performed to detect infected cells by staining with a human MAb against ZIKV E (envelope) protein (51) in addition to anti-Pax6 and anti β-III-tubulin (TUJ1) MAbs that stain neural progenitor cells and immature neurons, respectively. As shown in Fig. 7A, productive ZIKV infection was observed in untreated cell cultures with a peak of virus replication 8 days postinfection. Then, virus production started to decrease progressively due to the strong virus-induced cytopathic effect as shown by indirect immunofluorescence at days 12 and 14 postinfection (Fig. 7B). In contrast, when cells were incubated with heparin prior to infection, significantly lower levels of infectious virions were detected in the culture supernatant of treated cells than of controls as early as 1 day up to day 7 postinfection, and the peak of virus replication was delayed to 12 days postinfection (Fig. 7A). The incubation of cells with heparin after infection (posttreatment) was less effective than the pretreatment; no inhibition of viral replication was detected 24 h postinfection, although the kinetics of viral replication were delayed, and significantly lower levels of viral titers were obtained compared to control untreated cultures (Fig. 7A). The virus-positive cells were lower in pre- and post-heparin-treated cells than in untreated cells at day 7 postinfection, consistent with the inhibition of viral replication (Fig. 7B). At day 12 postinfection, the virus-induced cytopathic effect eliminated most of the cells in the untreated cultures, whereas heparin pre- or posttreatment prevented cell death. At the end of the differentiation period, i.e., day 14, TUJI-positive cells (expressing a marker of neuron differentiation) increased in the uninfected untreated cultures compared to those on day 12 when Pax6 staining, a neural progenitor marker, was still abundant. In the infected conditions, most of the cells were dead, irrespective of the heparin treatment (Fig. 7B).

Thus, heparin treatment partially preserved differentiation of hiPSC-NPCs into neurons regardless of whether it was added before or after infection. This effect lasted at least up to day 12 postinfection by delaying the virus-induced cytopathic effects.

### Repeated heparin additions to hiPSC-NPCs allowed their differentiation into neuroglial cells in the presence of persistent ZIKV infection.

**FIG 8:**
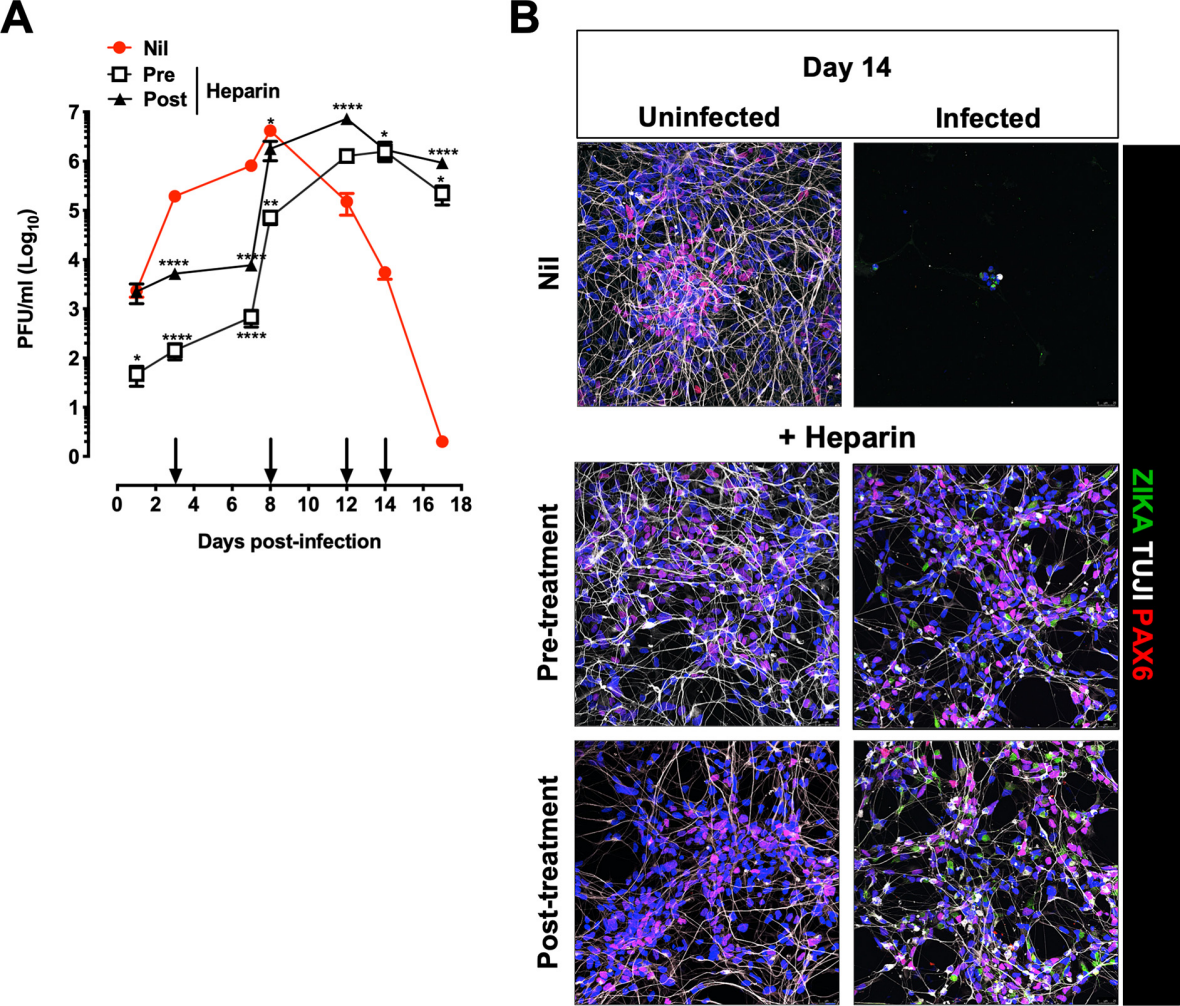
Kinetics of ZIKV replication in hiPSC-NPCs treated with multiple doses of heparin. (A) hiPSC-NPCs were differentiated for 2 weeks. Cells were treated with heparin either before (pre) PRVABC59 infection (MOI, 0.001) or after (post) infection. Heparin was added every 3 days (black arrows). Virion release in the supernatant was quantified at different time points by PFA. The graph represents the mean ± SD of 3 independent experiments. One-way ANOVA with Bonferroni correction was used in each time point. *, Statistical comparison among groups (*, *P* < 0.05; **, *P* < 0.01; ****, *P* < 0.0001). (B) Cells were fixed after 14 days postinfection and stained for ZIKV with the anti-E antibody (ZIKA-green), neurons with beta tubulin III (TUJ1-white), and PAX6 (red). Blue channel is DAPI staining for all cell types. Scale bar, 75 μm. Images were obtained using SP8 confocal microscope.

As a single treatment of hiPSC-NPCs with heparin delayed ZIKV cytopathic effect but did not reverse it, heparin was added to the cultures twice a week up to 14 days to favor the establishment of a condition of persistently productive infection in the absence of significant cytopathic effects. As shown in Fig. 8A, significantly lower levels of infectious virus were detected in the culture supernatant of treated cells than of controls as early as 1 day up to day 7 postinfection, and the kinetics of virus replication in heparin-treated cultures were delayed compared to those of untreated cultures; unlike what was observed with a single heparin treatment (Fig. 7A), however, in heparin-treated cells, the presence of infectious virus released in culture supernatants was extended at least up to 17 days postinfection when the experiments were terminated. Furthermore, readdition of heparin improved cell survival as shown in Fig. 8B. Nevertheless, heparin allowed the differentiation of hiPSC-NPCs into neurons by preventing virus-induced cell death despite productive ZIKV infection as also shown by staining with a human MAb against envelope E protein (Fig. 8B).

### Differentiation of infected hf-NPCs into neuroglial cells in the presence of heparin.

Unlike hiPSC-NPCs, hf-NPCs were induced to pan neuroglia differentiation on a Matrigel-coated surface and in NeuroCult NS-A differentiation medium for at least 14 days. hf-NPCs were then incubated with heparin either 1 h prior to or 4 h postinfection; after removal of the viral inoculum, fresh differentiation medium was replaced twice a week for the following 2 weeks.

**FIG 9:**
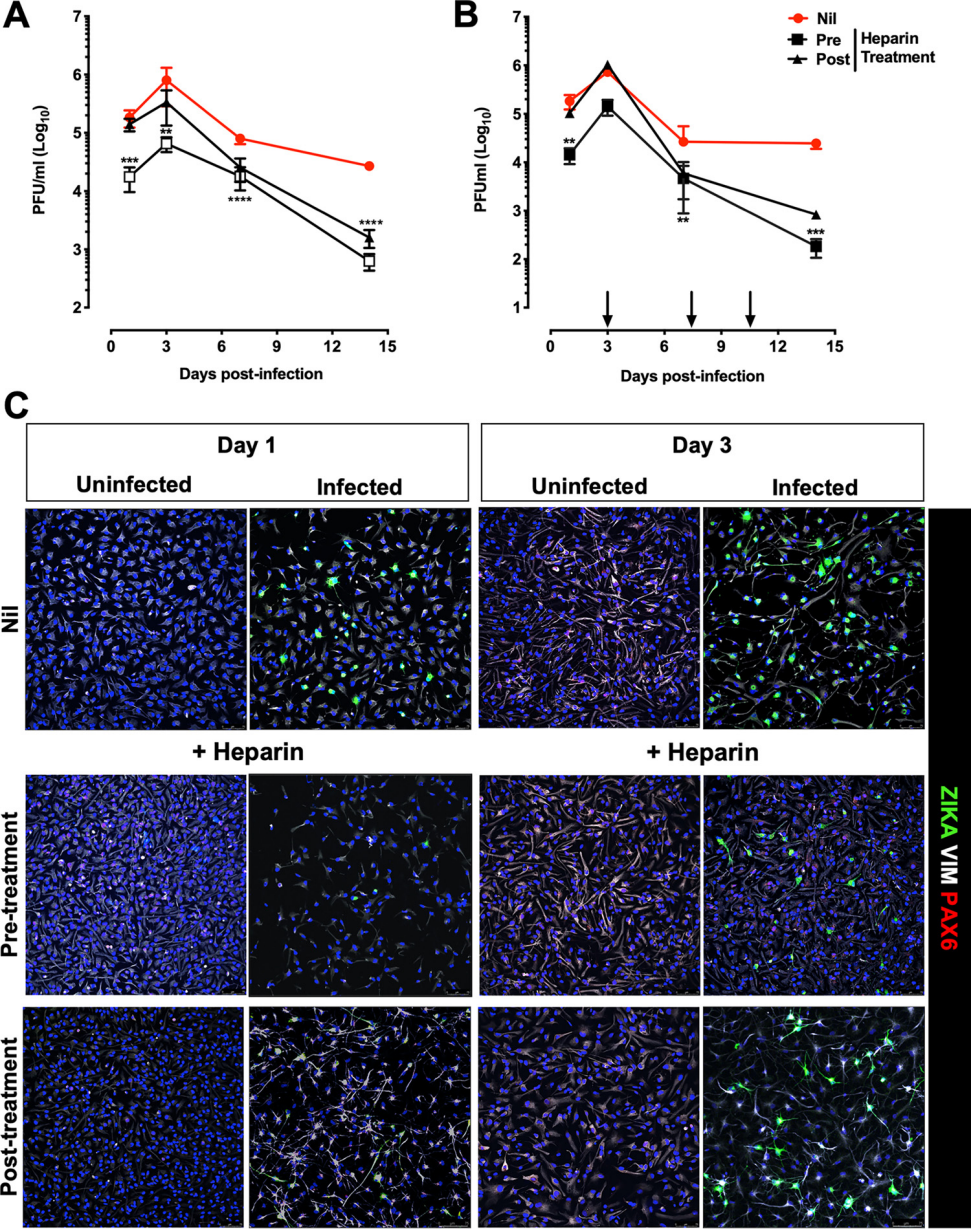

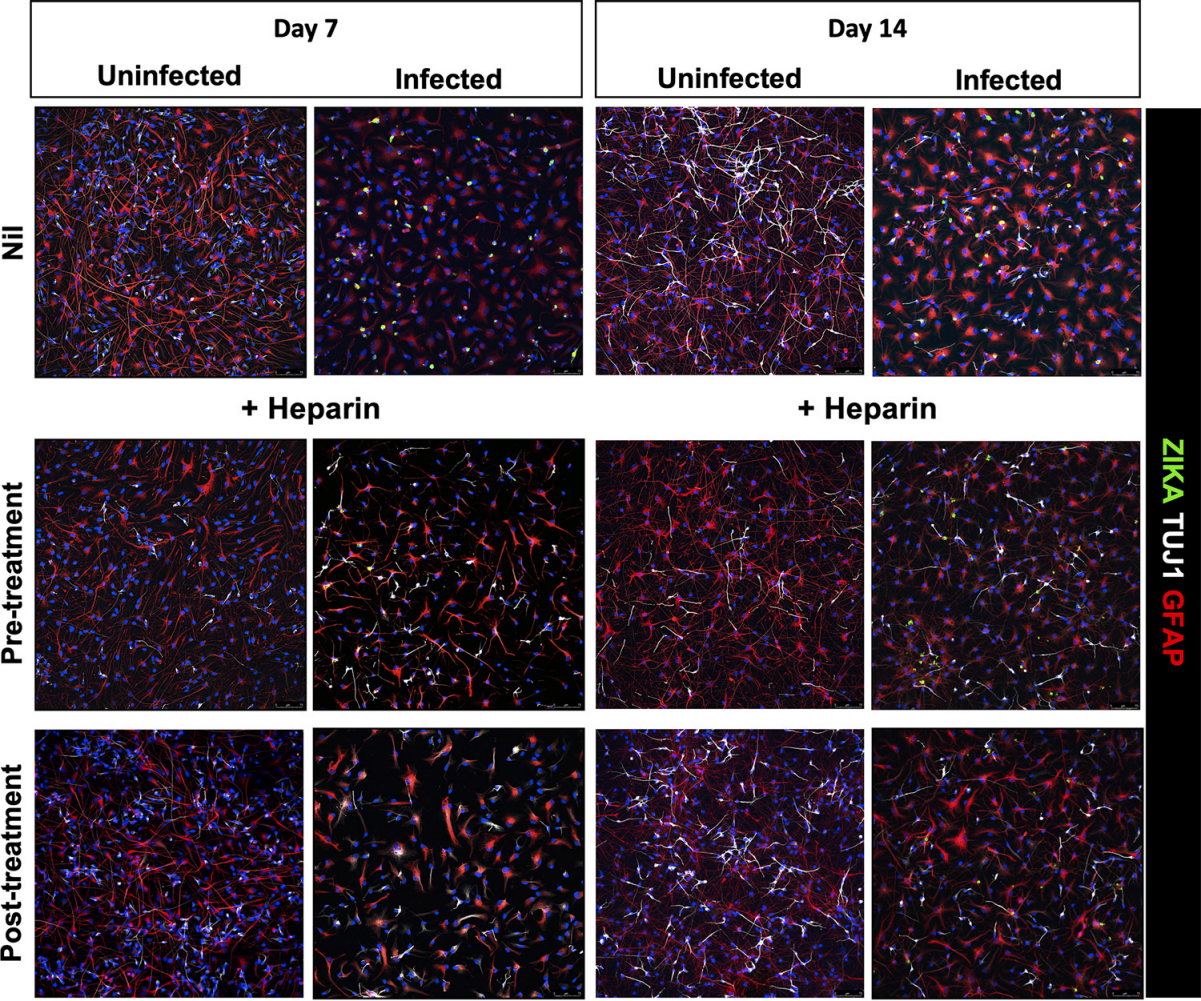
Kinetics of ZIKV replication in hf-NPCs treated with a single and multiple doses of heparin. (A) Infectious virus released in supernatant was measured by PFA. Cells were treated with heparin (100 μg/mL) either before (pre, 1 h) PRVABC59 infection (MOI, 5) or after (post, for 4 h) infection. Cells were then differentiated for 2 weeks in a differentiation medium. The graph represents the mean ± SD of 3 independent experiments. One-way ANOVA with Bonferroni correction was used. *, Statistical comparison among groups (**, *P* < 0.01; ***, *P* < 0.001; ****, *P* < 0.0001). (B) Cells were treated with heparin (100 μg/mL) prior to and after infection (1 h) with the PRVABC59 isolate at an MOI of 5. The differentiation medium was added, starting 4 h postinfection. Heparin was added every 3 days up to 14 days postinfection. Infectious titers were measured in the culture supernatants by PFA. Nil refers to untreated infected cultures, pre represents the treatment 1 h prior to infection, and post is the treatment 4 h postinfection. (C) Differentiated hf-NPCs were fixed at different time points; earlier time points (day 1 and 3 postinfection) were stained for ZIKV with the anti-E antibody (ZIKA-green), vimentin (VIM-white), and Pax6 (PAX6-red), whereas later time points (day 7 and 14 postinfection) were stained for ZIKV with the anti-E antibody (ZIKA-green), beta tubulin III (TUJ1-white), and GFAP (GFAP-red). The blue channel is DAPI staining for all cell types. Scale bar, 75 μm. Images were obtained using SP8 confocal microscope.

The pretreatment with heparin inhibited the amount of infectious virus released in the supernatant by ca. 1 log_10_ 24 h postinfection and, unlike hiPSC-NPCs, the kinetics of virus replication in cells incubated with heparin were like those of untreated cells. However, a significant reduction of infectious virus released in the supernatant was observed in heparin-pretreated cultures at day 1 and 3 postinfection (Fig. 9A), whereas the antiviral effects of posttreatment administration of heparin were delayed compared with those of pretreatment. Multiple additions of heparin did not change the kinetics of virus replication in either pre- or posttreatment conditions (Fig. 9B). Cells that were incubated only once with heparin were stained with vimentin, a marker poorly expressed in embryonic stem cells and switched-on early during their differentiation (52). As shown in Fig. 9C, vimentin expression in uninfected cells increased after 3 days of culture, whereas ZIKV infection did not alter its expression; heparin addition after infection accelerated hf-NPC differentiation.

**FIG 10:**
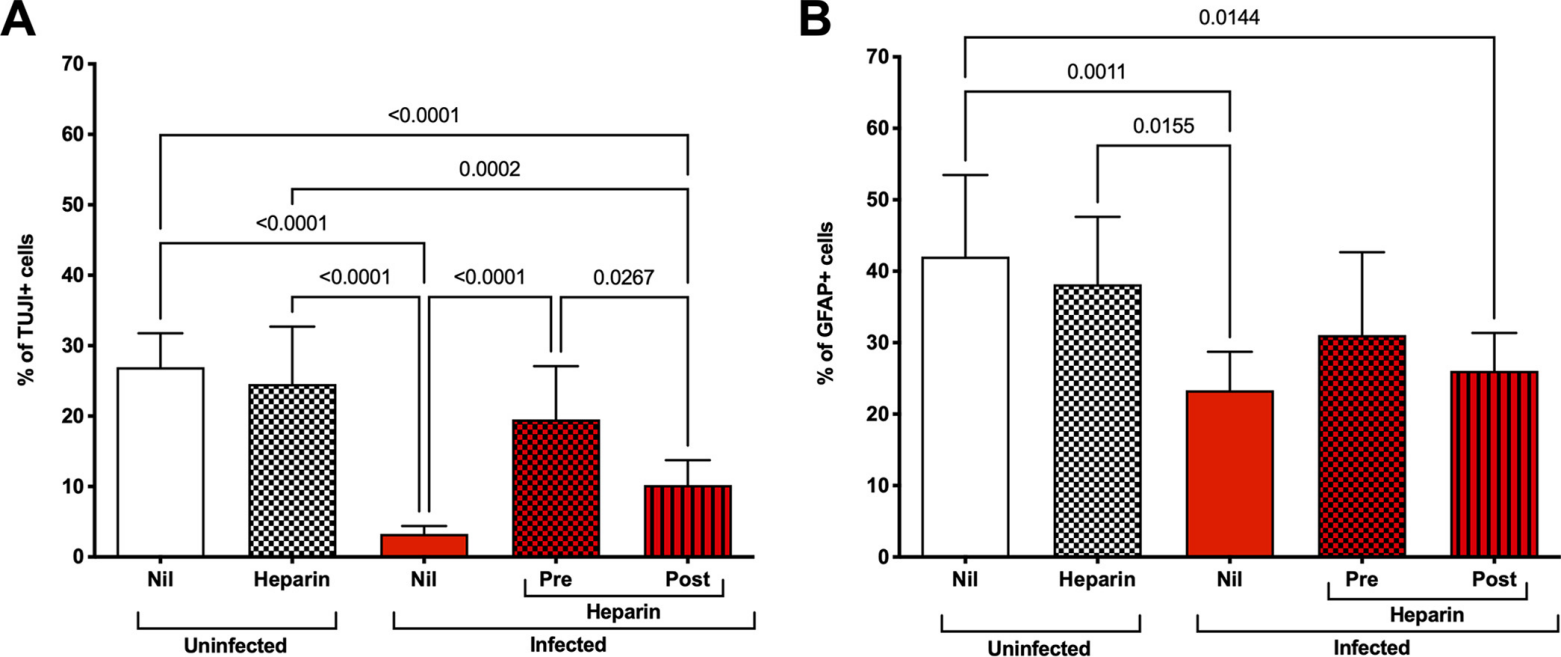
Quantification of neuroglial cells. (A) Quantification of neurons (TUJ1^+^ cells). hf-NPCs were treated with heparin either prior to or after infection with the PRVABC59-2015 isolate at an MOI of 5. After infection, cells were differentiated for 1 week. Cells were fixed after 7 days postinfection. (B) Quantification of astrocytes (GFAP^+^ cells). Three different images from 3 independent experiments were counted. Bars represent the percentage of neurons and astrocytes in the different conditions. *P* values were calculated by one-way ANOVA with the Bonferroni correction.

The differentiation status of the cells was examined after 7 days of differentiation (14 days postinfection) by staining the cell culture with TUJI and glial fibrillary acidic protein (GFAP), markers of differentiated neurons and astrocytes, respectively. Uninfected cells were positive for GFAP and TUJI after 7 days postdifferentiation, albeit the proportion of GFAP-positive cells was higher than that of TUJI-positive cells that became more visible 14 days postdifferentiation. At day 7, the GFAP signal was weak, and neurons were almost absent in infected untreated cells, whereas infected hf-NPCs treated with heparin prior to infection differentiated into neurons (TUJI-positive cells) and astrocytes (GFAP-positive cells) unlike untreated cells (Fig. 9C). At 14 days postinfection, neurons were well-represented in uninfected conditions, and a brilliant GFAP signal was detected, a phenotype previously correlated with cell activation status during inflammatory insults (53). Heparin-treated hNPC-derived astrocytes showed an intermediate phenotype, suggesting that heparin might protect from inflammation insult. The hf-NPC-derived astrocytes that survived showed a different morphology compared to those in uninfected conditions. As shown in Fig. 10A, a significant impairment in neuronal maturation was observed upon infection (~3% versus ~27% of neurons in infected and uninfected cultures, respectively), whereas the percentage of mature neural cells in infected cultures incubated with heparin was significantly higher (~19% with the pretreatment and ~10% with the posttreatment) and slightly differed in terms of cell number in comparison to uninfected cell culture (Fig. 10A). Differentiation of NPCs into astrocytes was observed in all conditions, although a moderate decrease from ~40% to ~20% of GFAP-positive cells in uninfected versus infected cultures, respectively, was detected. Heparin partially restored glial maturation in infected cell with an increase of ~30% of GFAP-positive cells in the pretreatment condition and ~25% in posttreatment condition (Fig. 10B).

Thus, heparin can prevent ZIKV-induced cytopathicity in hf-NPC and allow their differentiation into neural cells and astrocytes while exerting antiviral effects.

## DISCUSSION

Heparin protects hiPSC-NPCs from ZIKV-induced cell death regardless of whether they were grown in an adherent monolayer or as neurospheres (NS). In contrast to the lack of virus inhibition when cells were infected at high MOI, heparin inhibited the early steps of viral entry when cells were infected at low MOI. Furthermore, a single addition of heparin delayed ZIKV replication in hiPSC-NPCs that were committed to differentiate into neuroglial cells. Multiple additions of heparin, conversely, while preserving neuroglial differentiation and inhibiting virus-induced cell death prolonged ZIKV replication. Surprisingly, unlike what was observed with hiPSC-NPCs, when differentiating hf-NPCs were infected at high MOI in the presence of heparin, virus replication was significantly inhibited. In untreated cultures, virus persisted during their differentiation, while in heparin-treated cultures, ZIKV replication decreased and, significantly, hf-NPCs differentiated into neurons and glial cells. These results demonstrate the neuroprotective activity of heparin against the cytopathicity of ZIKV in different models of hNPC differentiation with variable effects in terms of interference with virus replication.

Similar to other members of the *Flaviviridae* family, ZIKV causes cytopathic infection of fetal neural progenitor cells that leads to defective neurogenesis (16, 54, 55). Several studies have reported apoptosis as a mechanism of cell death in ZIKV-infected hNPCs (27, 56); however, caspase 3-dependent apoptosis was also observed in adjacent cells without evidence of infection, as bystander cells are susceptible to the cytotoxic factors released during the cell death of productively infected cells (15). In addition, caspase 3-independent cytopathicity has also been reported in cells such as epithelial cells, primary skin fibroblasts and astrocytes (32). This mode of cell death was explained by massive vacuolization of the ER, which is the major intracellular site of ZIKV replication (32). The accumulation of ZIKV vacuoles triggers a cellular collapse termed paraptosis (32). Heparin-treated hNPCs were protected by ZIKV-induced apoptosis but also by virus-induced ER vacuolization in both hiPSC-NPCs and hf-NPCs grown either in adherence or as NS.

The protective mechanism of heparin probably relies on molecular interactions at the cell membrane level. In this regard, heparin is known to be a potent modulator of cellular receptors of growth factors (GFs), such as fibroblast growth factor (FGF), epidermal growth factor (EGF), and vascular endothelial growth factor (VEGF) (57). Among the GFs involved in neurogenesis, FGF-2, together with EGF, is essential for neuronal cell survival and maturation and plays a crucial role in central nervous system (CNS) development (58). The endogenous polysaccharide component, heparan sulfate (HS) of heparan sulfate proteoglycans (HSPGs), is the obligatory cofactor required to form functional, dimeric FGF/FGF receptor signaling complexes. Its activity is mimicked by heparin, which shares basic structural similarity, or by other polysaccharide components of other PGs, such as chondroitin sulfate (59, 60). Like HS, heparin can influence FGF-2 activity by interacting with its receptor or stabilizing it and preventing its degradation; this interaction is specific to Heparin/HS and is not general to other polysaccharide components of proteoglycans (61).

In the present study, we demonstrate the protective effect of heparin on virus-induced cell death of NS, a 3D system formed from spontaneous aggregations of hNPCs, a model suitable to investigate the consequences of infection under nonadherent conditions (17). Although brain organoids are the best 3D model for studying neurodevelopment (62), their preparation from hNPCs is complex and costly. Thus, NS that are permissive to ZIKV infection, which disrupts their spherical structure by virus-induced cytopathic effects (17), have become established as an alternative 3D model. We, therefore, tested whether heparin was effective in preventing cell death of ZIKV-infected NS derived from either hiPSC- or hf-NPCs. In this regard, hf-NPCs more closely mimic ZIKV tropism for the fetal developing brain *in utero* (40). ZIKV persistently replicated in hf-NPCs that were less susceptible to ZIKV cytopathic effects than hiPSC-NPCs, which is in agreement with a previous report (22). As expected, heparin strongly protected NS from ZIKV-induced cytopathic effects with both hiPSC-NPCs and hf-NPCs by preventing NS disruption and inhibited both apoptosis and necrosis. Unexpectedly, an antiviral activity of heparin was clearly demonstrated in NS obtained from hiPSC-NPCs that was not detected when hNPCs were maintained in the adherent condition of tissue culture. These discrepancies are likely explained by the lower MOI used to infect NS than that of adherent hNPCs. In addition, the MOI used to infect the NS was calculated taking into consideration the number of cells seeded prior to the formation of NS while the virus inoculum was added to the fully formed NS that were kept under nonadherent proliferating conditions for 3 days. Furthermore, virions could only encounter cells present on the more external layer of the sphere. While cell-to-cell spread of infection is more efficient than cell-free infection (63), the number of infectious particles that reached the center of the NS is likely to be lower than that of a simple monolayer in which all cells are exposed to the virus.

To characterize the antiviral heparin activity during ZIKV infection, we tested its activity during ZIKV entry using either a “high” MOI of 1 or a “low” MOI of 0.001 in the infection of adherent hiPSC-NPCs. Indeed, at low MOI, heparin exerted an antiviral activity against ZIKV infection in hiPSC-NPCs by interfering with viral attachment. In this regard, ZIKV as well as other members of the flavivirus family use negatively charged glycosaminoglycans (GAGs) to concentrate and gain access to surface receptors (64). Therefore, numerous polyanionic compounds, including heparin, compete with GAGs and inhibit enveloped-virus infection and replication *in vitro* (65–69). Using surface plasmon resonance, heparin has been shown to strongly bind to the ZIKV E protein with a dissociation constant (*K_D_*) of 443 nM, suggesting that ZIKV E-GAG interaction is likely to be driven by electrostatic interactions. Indeed, Kim and collaborators have recently reported that heparin could promote ZIKV infection in Vero cells at the MOI of 0.1 (70), albeit this viral-enhancing effect was observed with heparin concentrations of >100 μg/mL. In agreement with our results, heparin did not inhibit viral infection at high MOI, although it maintained protective activity by inhibiting ZIKV-induced cell death (70).

Since heparin exerted antiviral activity and protects against multiple forms of ZIKV-induced cell death, we considered the possibility that the cells maintained the ability to differentiate into neuroglial cells. To this end, ZIKV-infected hNPCs were differentiated into mature neuroglial cells. During differentiation in hiPSC-NPCs, heparin delayed the kinetics of viral replication, protected hNPCs from ZIKV-induced cell death, and allowed their differentiation into neurons and astrocytes. Interestingly, repeated additions of heparin sustained prolonged viral replication perhaps by blocking cell-to-cell spread while suppressing ZIKV-induced cytopathicity.

After the initial inhibition of virus replication, virus spreading was similar in both heparin-treated and control cultures, whereas a fraction of cells survived and continued to produce virus. It is unknown whether the initial reduction in the number of infected cells limited virus spreading to daughter cells during cell division and differentiation. Nevertheless, heparin is clearly endowed with beneficial effects by allowing ZIKV-infected hNPCs to differentiate into neuroglial cells.

In addition to the hiPSC-NPC model, we also exploited the hf-NPCs because they recapitulate the heterogeneity of neural precursor cells of the embryonic human brain (40). By inhibiting ZIKV replication and preventing virus-induced cell death, heparin-treated cultures showed a proportion of neurons and astrocytes, which was comparable to that of uninfected cultures. The absence of neurons in infected cultures could be caused by inflammatory mediators induced by ZIKV infection (71) influencing cell maturation that was nevertheless restored and maintained following incubation with heparin. These results demonstrate the neuroprotective activity of heparin against ZIKV cytopathicity in hNPCs infection.

The distinct behavior of hf-NPCs and hiPSC-NPCs could be explained by their diverse composition. Indeed, hiPSC-NPCs are homogeneous, whereas hf-NPCs consist of a highly heterogeneous mixture of stem cells and neuronal precursors at distinct early stages of differentiation (72). These features of hf-NPCs might resemble *in vivo* neurodevelopment more closely than that observed with hiPSC-derived NPCs, even though their limited availability and high heterogeneity represent a major drawback. Apart from differences between these two cell culture systems, the protective activity of heparin against ZIKV-induced cell death was strongly preserved in both models, although further investigation will be required to determine the mechanism(s) of protection.

Collectively, these results highlight the potential of heparin protective activity against neurotropic viruses. The unfractionated heparin (UFH) used in the present study is a highly sulfated polysaccharide endowed with anticoagulant activity. Given its high molecular weight (typically 12 to 15 kD), the proportion of UFH that can reach the fetus and protect the fetal brain from virus damage is likely to be low. Nevertheless, heparin is routinely chemically and enzymatically modified to obtain derivatives or fractions with low molecular weight (typically around 5 kDa), exhibiting increased bioavailability and more predictable anticoagulant activity and safety profile (73). It is highly likely that active fractions with low or no anticoagulant activity also reside in crude heparin, the precursor material to pharmaceutical UFH, a compound awaiting further exploitation (74). In addition to its anticoagulant activity, heparin can prevent tissue injuries at the fetal-maternal interface (75). For instance, heparin improves successful embryo implantation by suppressing natural killer cell cytotoxicity (76), prevents leukocyte adhesion/influx (77), antagonizes interferon-γ (IFN-γ) signaling (78), and modulates chemokine activity (79). Mouse models of ZIKV infection, particularly pregnant mice, will be instrumental in determining heparin beneficial effects on the fetal brain.

In conclusion, heparin, its derivatives (devoid of anticoagulant activity), or their analogues could be used as antiviral agents, especially in providing rapid countermeasures against present and future emerging viral diseases, such as the current SARS-CoV-2 infection (45).

## MATERIALS AND METHODS

### Ethics statement.

The study protocol was approved by the Ethical Committee of the IRCCS San Raffaele Hospital (Milan, Italy). Subjects participating in the study provided informed consent (Banca INSpe). This study conformed to the standards of the Declaration of Helsinki.

### Human iPSC-derived NPCs.

Fibroblasts were isolated from a skin biopsy of one healthy subject as described (80). Fibroblasts were reprogrammed into iPSCs by using the episomal Sendai virus approach (CytoTune-iPS 1.0 Sendai Kit; Life Technologies) to obtain hiPSCs. Cells were maintained in feeder-free conditions in mTeSR1 culture medium (Stem Cell Technologies) on Matrigel ES (MaES)-coated (Corning) plates and passaged using 0.5 mM EDTA. hNPCs were generated with some modifications of the protocol described in Reinhardt et al. (81). Briefly, colonies of hiPSCs grown on MaES were detached using dispase (Stemcell Technologies), maintained in human embryonic stem cell (hESC) medium without basic fibroblast growth factor (bFGF), and supplemented with 1 μM dorsomorphin (Stemgent), 3 μM CHIR99021 (Tocris), 10 μM SB-431542 (Miltenyi), and 0.5 μM purmorphamine (Alexis). Embryoid bodies (EBs) were formed by culturing cells in nontissue culture petri dishes (Greiner). On day 2, the medium was changed to N2B27 medium containing equal parts of Neurobasal (Invitrogen) and Dulbecco modified Eagle medium (DMEM)-F12 (Invitrogen) with 1:100 B27 supplement lacking vitamin A (Invitrogen), 1:200 N2 supplement (Invitrogen), 1% penicillin/streptomycin/glutamine (PSG) (Gibco), and the same small molecules as used above. On day 4, dorsomorphin and SB-431542 were withdrawn, while 150 μM ascorbic acid (AA) was added to the medium. On day 6, EBs were mechanically dissociated into smaller aggregates and seeded onto Matrigel growth factor reduced high concentration (Ma GFRH, Corning) coated 12-well plates (Corning). When hNPCs reached confluence, cells were detached with Accumax (Sigma) and replated (at least 1:5) in the presence of ROCK inhibitor (Calbiochem). After 3 passages, purmorphamine was replaced by 1 μM SAG (Calbiochem). hNPCs were expanded until passage 10 before infection and oligodendroglial differentiation. NS were obtained by maintaining single cells in nontissue culture petri dishes where they were grown as cell aggregates.

### Human fetal brain-isolated NPCs.

hf-NPCs are nonimmortalized human fetal neural precursor cells (named the BI-0194-008 cell line) obtained from a single human fetus at 10 to 12 weeks postconception (wpc) as reported previously (80). Human tissue was provided by “Banca Italiana - Fondazione IRCCS CA' GRANDA Ospedale Maggiore Policlinico di Milano.” Permission to use human fetal CNS tissue was granted by the ethical committee of the San Raffaele Hospital (approved on 13/06/2013) in agreement with the declaration of Helsinki and with the ethical guidelines of the European Network for Transplantation (NECTAR). The BI-0194-008 cell line is currently in use for the clinical trial EudraCT 2016-002020-86 (ClinicalTrials registration no. NCT03269071). Briefly, primary, growth factor-expanded hNPCs were obtained as heterogeneous culture of spherical cell aggregates, derived from the diencephalic and telencephalic regions. Cells were grown in suspension as spheres in flask in NeuroCult-XF proliferation medium human (Stemcell Technologies) with EGF and bFGF (10 ng/mL each; R&D Systems). Every 10 to 15 days, enzymatic dissociation of neurospheres (NS) with Accumax and reseeding (25,000 cells/cm^2^) were performed.

### Differentiation of hNPCs.

hiPSC-NPC-derived mature neuroglial cells were generated by an induction phase followed by a differentiation step with modification of a described protocol (50). Cells were seeded on Matrigel growth factor reduced (Ma GFR)-coated (Corning) 12-mm-diameter coverslips, and induction was allowed to occur in the presence of DMEM-F12 supplemented with N2 (1:50), B27 (1:50), minimal essential medium with nonessential amino acids (MEM-NEAA) (Gibco), PSG (Gibco), heparin (2 μg/mL), cAMP 1 μM (Sigma), and 10 ng/mL insulin growth factor-1 (IGF-1). Half of the medium was changed twice a week. After 7 days, the medium was replaced with Neurobasal medium, N2 (1:50), B27 (1:50), MEM-NEAA (Gibco), and PSG and supplemented with cAMP (1 μM), AA (150 μM), brain-derived neurotrophic factor (BDNF) (10 ng/mL), IGF-1 (10 ng/mL), and glial cell-derived neurotrophic factor (GDNF) (10 ng/mL).

To obtain hf-NPC pan-differentiation, spheres (passages 10 to 12) were dissociated in single cells and seeded on 12-mm (diameter) glass coverslips (7 × 10^4^/cells each) coated with Ma GFR (0.2 μg/mL). Cells were maintained in NeuroCult NS-A differentiation medium human (Stemcell Technologies) for up to 14 days.

### Viruses.

The following three virus isolates were used: the historical ZIKV strain (MR766) (European Virus Archive global [EVAg]), the Puerto Rico 2015-PRVABC59 obtained from the CDC (GenBank accession number KU501215), and the Brazilian 2016-INMI-1 (GenBank accession number KU991811) obtained from an Italian individual who travelled to Brazil in January 2016. Viral isolates were expanded in Vero cells and titrated by a plaque forming assay (PFA) as detailed further.

As an internal control, to obtain inactivated ZIKV (iZIKV), viral stocks were heat-inactivated at 65°C for 1 h. PFA was used to confirm complete inactivation.

### ZIKV early step infection assay.

hiPSC-NPCs were seeded at 2.5 × 10^5^ cells/well in 24-well flat-bottom plastic plates in 500 μL of complete medium. After 24 h, hNPCs were precooled at 4°C and then inoculated with the PRVABC59 isolate at an MOI of either 1 or 0.001 for 3 h. The unbound virus was removed by washing the cell cultures with cold medium (on ice), and the cell cultures were then warmed up to 37°C for 15 min to allow virus entry. The cell monolayers were then exposed to citrate buffer (pH 3.0) for 1 min to inactivate any virus that did not penetrate the target cells. Next, the cells were washed and overlaid with medium. Heparin was added either 1 h prior to ZIKV infection at 4°C or during the 15-min incubation at 37°C to determine whether heparin acted at the level of attachment or the level of entry, respectively (46). Three days after infection, supernatants were collected and stored at −80°C until determination of the infectious titer by PFA.

### Ultrastructural analysis.

Adherent hiPSC-NPCs were infected at the MOI of 1 with the PRVABC59 isolate. Three days after infection, cells were fixed in 4% formaldehyde and 2.5% glutaraldehyde in cacodylate buffer and incubated for 5 min at room temperature. The samples were then fixed with 2% osmium tetroxide in 2.5% glutaraldehyde in cacodylate buffer for 60 min. Monolayers were dehydrated in graded ethanol, washed in propylene oxide, and infiltrated for 12 h in a 1:1 mixture of propylene oxide and epoxide resin (Epon). Cells were then embedded in Epon and polymerized for 24 h at 60°C. Slices were cut with an ultramicrotome (Ultracut Uct; Leica), stained with uranyl acetate and lead citrate, and metaled. The ultrathin sections of infected hiPSC-NPCs were observed through transmission electron microscopy (Hitachi H7000).

### ZIKV infection of NS.

Both hiPSC- and hf-NPCs were grown spontaneously under rotation as NS after seeding for 3 days before infection. Porcine intestine mucosal heparin (Celsus Laboratories Inc.) was added 1 h prior to infection at a final concentration of 100 μg/mL. NS were infected with the Brazilian 2016-INMI-1 isolate at an MOI of 1. Viral supernatants were collected 3 and 6 days postinfection. Cell death was determined by measuring the levels of adenylate kinase activity in the culture supernatant (ToxiLight Bioassay, Lonza). The viral titers were determined by a PFA. Brightfield images were captured at 3 and 6 days postinfection to measure NS diameter by using ImageJ software (https://imagej.nih.gov/ij/). NS were transferred onto slides precoated with Ma GFR and fixed at 3 and 6 days postinfection for the evaluation of the efficiency of infection by immunofluorescence with specific antibodies.

### ZIKV infection of hiPSC-NPCs induced to differentiate into neuroglial cells.

hiPSC-NPCs were seeded into 24-well plates at a final concentration of 1 × 10^5^ cells/well. Twenty-four h postseeding, cells were infected with the PRVABC59 isolate at an MOI of 0.001 and incubated with heparin (100 μg/mL). Two distinct protocols were adopted to test heparin effects on differentiation of hNPCs into neuroglial cells. In the pretreatment protocol, cells were incubated with heparin 1 h prior to infection. After 4 h, the virus inoculum was removed and induction medium supplemented with cAMP, and IGF-1 was added for 1 week, followed by replacement with differentiation medium for the following 3 weeks. In the posttreatment protocol, cells were infected for 4 h and then the virus inoculum was removed and heparin was added for 1 h. Culture medium was replaced with the induction medium supplemented with cAMP and IGF-1 and changed every 3 days. After 1 week, the induction medium was replaced with the differentiation medium for the following 3 weeks. The differentiation medium was replaced with fresh medium every 3 days. Supernatants were harvested at different time points to determine the infectious titers, cells were fixed side-by-side, and cell differentiation was determined during infection with specific antibody staining.

### ZIKV infection of hf-NPCs induced to differentiate into neuroglial cells.

hf-NPCs were placed in a 15-mL Falcon tube at a final concentration of 7 × 10^4^ cells/well. The PRVABC59 isolate was added at an MOI of 5, and heparin was used at 100 μg/mL. As described above, in the pretreatment protocol, cells were incubated with heparin 1 h prior to infection. After 4 h, cells were centrifuged, resuspended in NeuroCult NS-A differentiation medium (Stemcell), and seeded into 24-well plates. Cultures were maintained for 14 days. In the posttreatment protocol, cells were infected for 4 h and then centrifuged, resuspended in NeuroCult NS-A differentiation medium (Stemcell), and incubated with heparin for 1 h. The differentiation medium was replaced with fresh medium every 3 days up to 14 days. Supernatants were harvested at different time points to determine the infectious titers, cells were fixed side-by-side, and cell differentiation was determined during infection with specific antibody staining.

### Immunofluorescence and image capture.

NS were fixed using 4% paraformaldehyde (PFA) for 10 min, washed three times with phosphate-buffered saline (PBS) and incubated for 1 h with blocking solution PBS-0.5% Triton X-100, 5% donkey serum solution. NS were stained with mouse monoclonal Ab (MAb) anti-double-stranded RNA (dsRNA) (Jena Bioscience), rabbit anti-cl-CASP3 (Cell Signaling), and goat anti-vimentin (Sigma) polyclonal antibodies overnight in blocking solution. After three washings with PBS-0.1% Triton X-100, Alexa Fluor-conjugated secondary antibodies were incubated for 2 h in blocking solution at room temperature and then cells were washed three times with PBS-0.1% Triton X-100. DAPI (4′,6-diamidino-2-phenylindole) was used to stain the nuclei. NS and cells were visualized and acquired using a Leica SP8 confocal microscope (Alembic Facilities; https://research.hsr.it/en/core-facilities/alembic.html). Merged images were then generated using ImageJ software.

Differentiated hNPCs were fixed using 4% PFA for 10 min, washed 3 times with PBS, and incubated for 1 h with blocking solution containing 0.5% Triton X-100 and 5% donkey serum in PBS. Cells were then stained with specific primary antibodies, in particular, the human monoclonal antibody ZKA190-rIgG1 against the flavivirus E protein (51), the chicken anti-neuron-specific class III beta-tubulin (TUJ1; BioLegend), rabbit anti-glial fibrillary acidic protein (GFAP) (Dako), and the rabbit anti-PAX6 (BioLegend) polyclonal antibodies overnight in blocking solution. After 3 washings with PBS-0.1% Triton X-100, Alexa Fluor-conjugated secondary antibodies were incubated for 2 h in blocking solution at room temperature and washed 3 times with PBS-0.1% Triton X-100 solution. DAPI was used to stain the nuclei.

### Plaque forming assay.

Vero cells (1.2 × 10^6^) were seeded in 6-well culture plates; 24 h later, 10-fold dilutions of virus-containing supernatants were prepared in culture medium supplemented with 1% heat-inactivated fetal bovine serum (FBS), and 1 mL of each dilution was added to the cells. The plates were incubated for 4 h at 37°C. Unabsorbed virus was removed, and 2 mL of culture medium supplemented with 1% methylcellulose (Sigma) was added to each well, followed by an incubation at 37°C for 6 days. The methylcellulose overlay was removed, and the cells were stained with 1% crystal violet in 70% methanol. Plaques were counted and expressed as plaque-forming units per milliliter (PFU/mL) as published (31).

### Necrotic cell death detection assay.

ToxiLight bioassay (Lonza) was used to measure the adenylate kinase (AK) activity in culture supernatants as a marker of cell death. Briefly, 10-μL samples of culture supernatant were transferred on black 96-well half-area plates (Costar). Fifty microliters of detection reagent was added to each well, and the plate was incubated for 10 min at room temperature. Luminescence was measured in a Mithras LB940 microplate reader (Berthold Technologies), and the results were expressed as relative luminescent units (RLU).

### HMGB1 ELISA.

The HMGB1 levels were determined in sample supernatants by a commercially available enzyme-linked immunosorbent assay (ELISA) kit (HMGB1 ELISA kit II; Shino-Test Corporation) according to the manufacturer’s protocol. Briefly, the wells of the microtiter strips were coated with purified anti-HMGB1 MAb. HMGB1 in the sample binds specifically to the immobilized antibody and is recognized by a second enzyme-marked antibody. After substrate reaction, the HMGB1 concentration was determined by the color intensity.

### Statistical analysis.

Prism GraphPad software v. 8.0 (https://www.graphpad.com/) was used for all statistical analyses. Fisher’s exact test was used to compare the distribution of cells with and without vacuoles. Comparison between two groups was determined with a Student's *t* test. Comparison among groups was performed using one-way analysis of variance (ANOVA) and Bonferroni’s multiple comparison test.

## References

[1] Musso D, Gubler DJ. 2016. Zika virus. Clin Microbiol Rev 29:487–524. 10.1128/CMR.00072-15. 27029595 PMC4861986

[2] D'Ortenzio E, Matheron S, Yazdanpanah Y, de Lamballerie X, Hubert B, Piorkowski G, Maquart M, Descamps D, Damond F, Leparc-Goffart I. 2016. Evidence of sexual transmission of Zika virus. N Engl J Med 374:2195–2198. 10.1056/NEJMc1604449. 27074370

[3] Moreira J, Lamas CC, Siqueira A. 2016. Sexual transmission of Zika virus: implications for clinical care and public health policy. Clin Infect Dis 63:141–142. 10.1093/cid/ciw211. 27048746

[4] Motta IJ, Spencer BR, Cordeiro da Silva SG, Arruda MB, Dobbin JA, Gonzaga YB, Arcuri IP, Tavares RC, Atta EH, Fernandes RF, Costa DA, Ribeiro LJ, Limonte F, Higa LM, Voloch CM, Brindeiro RM, Tanuri A, Ferreira OC, Jr. 2016. Evidence for transmission of Zika virus by platelet transfusion. N Engl J Med 375:1101–1103. 10.1056/NEJMc1607262. 27532622

[5] Dick GW. 1952. Zika virus. II. Pathogenicity and physical properties. Trans R Soc Trop Med Hyg 46:521–534. 10.1016/0035-9203(52)90043-6. 12995441

[6] Haddow AD, Schuh AJ, Yasuda CY, Kasper MR, Heang V, Huy R, Guzman H, Tesh RB, Weaver SC. 2012. Genetic characterization of Zika virus strains: geographic expansion of the Asian lineage. PLoS Negl Trop Dis 6:e1477. 10.1371/journal.pntd.0001477. 22389730 PMC3289602

[7] Duffy MR, Chen TH, Hancock WT, Powers AM, Kool JL, Lanciotti RS, Pretrick M, Marfel M, Holzbauer S, Dubray C, Guillaumot L, Griggs A, Bel M, Lambert AJ, Laven J, Kosoy O, Panella A, Biggerstaff BJ, Fischer M, Hayes EB. 2009. Zika virus outbreak on Yap Island, Federated States of Micronesia. N Engl J Med 360:2536–2543. 10.1056/NEJMoa0805715. 19516034

[8] Cao-Lormeau VM, Roche C, Teissier A, Robin E, Berry AL, Mallet HP, Sall AA, Musso D. 2014. Zika virus, French Polynesia, South Pacific, 2013. Emerg Infect Dis 20:1085–1086. 10.3201/eid2006.140138. 24856001 PMC4036769

[9] Brasil P, Pereira JP, Moreira ME, Ribeiro Nogueira RM, Damasceno L, Wakimoto M, Rabello RS, Valderramos SG, Halai U-A, Salles TS, Zin AA, Horovitz D, Daltro P, Boechat M, Raja Gabaglia C, Carvalho de Sequeira P, Pilotto JH, Medialdea-Carrera R, Cotrim da Cunha D, Abreu de Carvalho LM, Pone M, Machado Siqueira A, Calvet GA, Rodrigues Baião AE, Neves ES, Nassar de Carvalho PR, Hasue RH, Marschik PB, Einspieler C, Janzen C, Cherry JD, Bispo de Filippis AM, Nielsen-Saines K. 2016. Zika virus infection in pregnant women in Rio de Janeiro. N Engl J Med 375:2321–2334. 10.1056/NEJMoa1602412. 26943629 PMC5323261

[10] Driggers RW, Ho CY, Korhonen EM, Kuivanen S, Jaaskelainen AJ, Smura T, Rosenberg A, Hill DA, DeBiasi RL, Vezina G, Timofeev J, Rodriguez FJ, Levanov L, Razak J, Iyengar P, Hennenfent A, Kennedy R, Lanciotti R, du Plessis A, Vapalahti O. 2016. Zika virus infection with prolonged maternal viremia and fetal brain abnormalities. N Engl J Med 374:2142–2151. 10.1056/NEJMoa1601824. 27028667

[11] Hazin AN, Poretti A, Cruz DD, Tenorio M, van der Linden A, Pena LJ, Brito C, Gil LH, Miranda-Filho DB, Marques ET, Martelli CM, Alves JG, Huisman TA. 2016. Computed tomographic findings in microcephaly associated with Zika virus. N Engl J Med 374:2193–2195. 10.1056/NEJMc1603617. 27050112

[12] Besnard M, Eyrolle-Guignot D, Guillemette-Artur P, Lastere S, Bost-Bezeaud F, Marcelis L, Abadie V, Garel C, Moutard ML, Jouannic JM, Rozenberg F, Leparc-Goffart I, Mallet HP. 2016. Congenital cerebral malformations and dysfunction in fetuses and newborns following the 2013 to 2014 Zika virus epidemic in French Polynesia. Euro Surveill 21:30181. 10.2807/1560-7917.ES.2016.21.13.30181. 27063794

[13] Calvet G, Aguiar RS, Melo AS, Sampaio SA, de Filippis I, Fabri A, Araujo ES, de Sequeira PC, de Mendonca MC, de Oliveira L, Tschoeke DA, Schrago CG, Thompson FL, Brasil P, Dos Santos FB, Nogueira RM, Tanuri A, de Filippis AM. 2016. Detection and sequencing of Zika virus from amniotic fluid of fetuses with microcephaly in Brazil: a case study. Lancet Infect Dis 16:653–660. 10.1016/S1473-3099(16)00095-5. 26897108

[14] Mlakar J, Korva M, Tul N, Popovic M, Poljsak-Prijatelj M, Mraz J, Kolenc M, Resman Rus K, Vesnaver Vipotnik T, Fabjan Vodusek V, Vizjak A, Pizem J, Petrovec M, Avsic Zupanc T. 2016. Zika virus associated with microcephaly. N Engl J Med 374:951–958. 10.1056/NEJMoa1600651. 26862926

[15] Ho CY, Ames HM, Tipton A, Vezina G, Liu JS, Scafidi J, Torii M, Rodriguez FJ, du Plessis A, DeBiasi RL. 2017. Differential neuronal susceptibility and apoptosis in congenital Zika virus infection. Ann Neurol 82:121–127. 10.1002/ana.24968. 28556287 PMC13040465

[16] Tang H, Hammack C, Ogden SC, Wen Z, Qian X, Li Y, Yao B, Shin J, Zhang F, Lee EM, Christian KM, Didier RA, Jin P, Song H, Ming GL. 2016. Zika virus infects human cortical neural progenitors and attenuates their growth. Cell Stem Cell 18:587–590. 10.1016/j.stem.2016.02.016. 26952870 PMC5299540

[17] Garcez PP, Loiola EC, Madeiro da Costa R, Higa LM, Trindade P, Delvecchio R, Nascimento JM, Brindeiro R, Tanuri A, Rehen SK. 2016. Zika virus impairs growth in human neurospheres and brain organoids. Science 352:816–818. 10.1126/science.aaf6116. 27064148

[18] Souza BS, Sampaio GL, Pereira CS, Campos GS, Sardi SI, Freitas LA, Figueira CP, Paredes BD, Nonaka CK, Azevedo CM, Rocha VP, Bandeira AC, Mendez-Otero R, Dos Santos RR, Soares MB. 2016. Zika virus infection induces mitosis abnormalities and apoptotic cell death of human neural progenitor cells. Sci Rep 6:39775. 10.1038/srep39775. 28008958 PMC5180086

[19] Onorati M, Li Z, Liu F, Sousa AMM, Nakagawa N, Li M, Dell'Anno MT, Gulden FO, Pochareddy S, Tebbenkamp ATN, Han W, Pletikos M, Gao T, Zhu Y, Bichsel C, Varela L, Szigeti-Buck K, Lisgo S, Zhang Y, Testen A, Gao XB, Mlakar J, Popovic M, Flamand M, Strittmatter SM, Kaczmarek LK, Anton ES, Horvath TL, Lindenbach BD, Sestan N. 2016. Zika virus disrupts phospho-TBK1 localization and mitosis in human neuroepithelial stem cells and radial glia. Cell Rep 16:2576–2592. 10.1016/j.celrep.2016.08.038. 27568284 PMC5135012

[20] Nowakowski TJ, Pollen AA, Di Lullo E, Sandoval-Espinosa C, Bershteyn M, Kriegstein AR. 2016. Expression analysis highlights AXL as a candidate Zika virus entry receptor in neural stem cells. Cell Stem Cell 18:591–596. 10.1016/j.stem.2016.03.012. 27038591 PMC4860115

[21] Dang J, Tiwari SK, Lichinchi G, Qin Y, Patil VS, Eroshkin AM, Rana TM. 2016. Zika virus depletes neural progenitors in human cerebral organoids through activation of the innate immune receptor TLR3. Cell Stem Cell 19:258–265. 10.1016/j.stem.2016.04.014. 27162029 PMC5116380

[22] Hanners NW, Eitson JL, Usui N, Richardson RB, Wexler EM, Konopka G, Schoggins JW. 2016. Western Zika virus in human fetal neural progenitors persists long term with partial cytopathic and limited immunogenic effects. Cell Rep 15:2315–2322. 10.1016/j.celrep.2016.05.075. 27268504 PMC5645151

[23] Yoon KJ, Song G, Qian X, Pan J, Xu D, Rho HS, Kim NS, Habela C, Zheng L, Jacob F, Zhang F, Lee EM, Huang WK, Ringeling FR, Vissers C, Li C, Yuan L, Kang K, Kim S, Yeo J, Cheng Y, Liu S, Wen Z, Qin CF, Wu Q, Christian KM, Tang H, Jin P, Xu Z, Qian J, Zhu H, Song H, Ming GL. 2017. Zika-virus-encoded NS2A disrupts mammalian cortical neurogenesis by degrading adherens junction proteins. Cell Stem Cell 21:349–358. 10.1016/j.stem.2017.07.014. 28826723 PMC5600197

[24] Morrison TE, Diamond MS. 2017. Animal models of Zika virus infection, pathogenesis, and immunity. J Virol 91:e00009-17. 10.1128/JVI.00009-17. 28148798 PMC5375682

[25] Miner JJ, Cao B, Govero J, Smith AM, Fernandez E, Cabrera OH, Garber C, Noll M, Klein RS, Noguchi KK, Mysorekar IU, Diamond MS. 2016. Zika virus infection during pregnancy in mice causes placental damage and fetal demise. Cell 165:1081–1091. 10.1016/j.cell.2016.05.008. 27180225 PMC4874881

[26] Cugola FR, Fernandes IR, Russo FB, Freitas BC, Dias JLM, Guimarães KP, Benazzato C, Almeida N, Pignatari GC, Romero S, Polonio CM, Cunha I, Freitas CL, Brandão WN, Rossato C, Andrade DG, Faria DdP, Garcez AT, Buchpigel CA, Braconi CT, Mendes E, Sall AA, Zanotto PMdA, Peron JPS, Muotri AR, Beltrão-Braga PCB. 2016. The Brazilian Zika virus strain causes birth defects in experimental models. Nature 534:267–271. 10.1038/nature18296. 27279226 PMC4902174

[27] Hamel R, Dejarnac O, Wichit S, Ekchariyawat P, Neyret A, Luplertlop N, Perera-Lecoin M, Surasombatpattana P, Talignani L, Thomas F, Cao-Lormeau VM, Choumet V, Briant L, Despres P, Amara A, Yssel H, Misse D. 2015. Biology of Zika virus infection in human skin cells. J Virol 89:8880–8896. 10.1128/JVI.00354-15. 26085147 PMC4524089

[28] Vicenzi E, Pagani I, Ghezzi S, Taylor SL, Rudd TR, Lima MA, Skidmore MA, Yates EA. 2018. Subverting the mechanisms of cell death: flavivirus manipulation of host cell responses to infection. Biochem Soc Trans 46:609–617. 10.1042/BST20170399. 29678952

[29] Cortese M, Goellner S, Acosta EG, Neufeldt CJ, Oleksiuk O, Lampe M, Haselmann U, Funaya C, Schieber N, Ronchi P, Schorb M, Pruunsild P, Schwab Y, Chatel-Chaix L, Ruggieri A, Bartenschlager R. 2017. Ultrastructural characterization of Zika virus replication factories. Cell Rep 18:2113–2123. 10.1016/j.celrep.2017.02.014. 28249158 PMC5340982

[30] Gladwyn-Ng I, Cordon-Barris L, Alfano C, Creppe C, Couderc T, Morelli G, Thelen N, America M, Bessieres B, Encha-Razavi F, Bonniere M, Suzuki IK, Flamand M, Vanderhaeghen P, Thiry M, Lecuit M, Nguyen L. 2018. Stress-induced unfolded protein response contributes to Zika virus-associated microcephaly. Nat Neurosci 21:63–71. 10.1038/s41593-017-0038-4. 29230053

[31] Volpi VG, Pagani I, Ghezzi S, Iannacone M, D'Antonio M, Vicenzi E. 2018. Zika virus replication in dorsal root ganglia explants from interferon receptor1 knockout mice causes myelin degeneration. Sci Rep 8:10166. 10.1038/s41598-018-28257-5. 29976926 PMC6033858

[32] Monel B, Compton AA, Bruel T, Amraoui S, Burlaud-Gaillard J, Roy N, Guivel-Benhassine F, Porrot F, Genin P, Meertens L, Sinigaglia L, Jouvenet N, Weil R, Casartelli N, Demangel C, Simon-Loriere E, Moris A, Roingeard P, Amara A, Schwartz O. 2017. Zika virus induces massive cytoplasmic vacuolization and paraptosis-like death in infected cells. EMBO J 36:1653–1668. 10.15252/embj.201695597. 28473450 PMC5470047

[33] Sperandio S, Poksay KS, Schilling B, Crippen D, Gibson BW, Bredesen DE. 2010. Identification of new modulators and protein alterations in non-apoptotic programmed cell death. J Cell Biochem 111:1401–1412. 10.1002/jcb.22870. 20830744 PMC5668132

[34] Luo Y, Chihara Y, Fujimoto K, Sasahira T, Kuwada M, Fujiwara R, Fujii K, Ohmori H, Kuniyasu H. 2013. High mobility group box 1 released from necrotic cells enhances regrowth and metastasis of cancer cells that have survived chemotherapy. Eur J Cancer 49:741–751. 10.1016/j.ejca.2012.09.016. 23040637

[35] Scaffidi P, Misteli T, Bianchi ME. 2002. Release of chromatin protein HMGB1 by necrotic cells triggers inflammation. Nature 418:191–195. 10.1038/nature00858. 12110890

[36] McCauley MJ, Zimmerman J, Maher LJ, III, Williams MC. 2007. HMGB binding to DNA: single and double box motifs. J Mol Biol 374:993–1004. 10.1016/j.jmb.2007.09.073. 17964600 PMC2117627

[37] Lange SS, Mitchell DL, Vasquez KM. 2008. High mobility group protein B1 enhances DNA repair and chromatin modification after DNA damage. Proc Natl Acad Sci USA 105:10320–10325. 10.1073/pnas.0803181105. 18650382 PMC2492475

[38] Bianchi ME, Crippa MP, Manfredi AA, Mezzapelle R, Rovere Querini P, Venereau E. 2017. High-mobility group box 1 protein orchestrates responses to tissue damage via inflammation, innate and adaptive immunity, and tissue repair. Immunol Rev 280:74–82. 10.1111/imr.12601. 29027228

[39] Ghezzi S, Cooper L, Rubio A, Pagani I, Capobianchi MR, Ippolito G, Pelletier J, Meneghetti MCZ, Lima MA, Skidmore MA, Broccoli V, Yates EA, Vicenzi E. 2017. Heparin prevents Zika virus induced-cytopathic effects in human neural progenitor cells. Antiviral Res 140:13–17. 10.1016/j.antiviral.2016.12.023. 28063994 PMC7113768

[40] Stein JL, de la Torre-Ubieta L, Tian Y, Parikshak NN, Hernandez IA, Marchetto MC, Baker DK, Lu D, Hinman CR, Lowe JK, Wexler EM, Muotri AR, Gage FH, Kosik KS, Geschwind DH. 2014. A quantitative framework to evaluate modeling of cortical development by neural stem cells. Neuron 83:69–86. 10.1016/j.neuron.2014.05.035. 24991955 PMC4277209

[41] Raucci A, Palumbo R, Bianchi ME. 2007. HMGB1: a signal of necrosis. Autoimmunity 40:285–289. 10.1080/08916930701356978. 17516211

[42] Kobolak J, Teglasi A, Bellak T, Janstova Z, Molnar K, Zana M, Bock I, Laszlo L, Dinnyes A. 2020. Human induced pluripotent stem cell-derived 3D-neurospheres are suitable for neurotoxicity screening. Cells 9:1122. 10.3390/cells9051122. PMC7290365 32369990

[43] Dmitriev RI, Zhdanov AV, Nolan YM, Papkovsky DB. 2013. Imaging of neurosphere oxygenation with phosphorescent probes. Biomaterials 34:9307–9317. 10.1016/j.biomaterials.2013.08.065. 24016849

[44] Vicenzi E, Canducci F, Pinna D, Mancini N, Carletti S, Lazzarin A, Bordignon C, Poli G, Clementi M. 2004. Coronaviridae and SARS-associated coronavirus strain HSR1. Emerg Infect Dis 10:413–418. 10.3201/eid1003.030683. 15109406 PMC3322807

[45] Mycroft-West CJ, Su D, Pagani I, Rudd TR, Elli S, Gandhi NS, Guimond SE, Miller GJ, Meneghetti MCZ, Nader HB, Li Y, Nunes QM, Procter P, Mancini N, Clementi M, Bisio A, Forsyth NR, Ferro V, Turnbull JE, Guerrini M, Fernig DG, Vicenzi E, Yates EA, Lima MA, Skidmore MA. 2020. Heparin inhibits cellular invasion by SARS-CoV-2: structural dependence of the interaction of the spike S1 receptor-binding domain with heparin. Thromb Haemost 120:1700–1715. 10.1055/s-0040-1721319. 33368089 PMC7869224

[46] Cheshenko N, Herold BC. 2002. Glycoprotein B plays a predominant role in mediating herpes simplex virus type 2 attachment and is required for entry and cell-to-cell spread. J Gen Virol 83:2247–2255. 10.1099/0022-1317-83-9-2247. 12185280

[47] Noctor SC, Martinez-Cerdeno V, Kriegstein AR. 2008. Distinct behaviors of neural stem and progenitor cells underlie cortical neurogenesis. J Comp Neurol 508:28–44. 10.1002/cne.21669. 18288691 PMC2635107

[48] Morshead CM, Reynolds BA, Craig CG, McBurney MW, Staines WA, Morassutti D, Weiss S, van der Kooy D. 1994. Neural stem cells in the adult mammalian forebrain: a relatively quiescent subpopulation of subependymal cells. Neuron 13:1071–1082. 10.1016/0896-6273(94)90046-9. 7946346

[49] Morest DK, Silver J. 2003. Precursors of neurons, neuroglia, and ependymal cells in the CNS: what are they? Where are they from? How do they get where they are going? Glia 43:6–18. 10.1002/glia.10238. 12761861

[50] Muratore CR, Srikanth P, Callahan DG, Young-Pearse TL. 2014. Comparison and optimization of hiPSC forebrain cortical differentiation protocols. PLoS One 9:e105807. 10.1371/journal.pone.0105807. 25165848 PMC4148335

[51] Wang J, Bardelli M, Espinosa DA, Pedotti M, Ng TS, Bianchi S, Simonelli L, Lim EXY, Foglierini M, Zatta F, Jaconi S, Beltramello M, Cameroni E, Fibriansah G, Shi J, Barca T, Pagani I, Rubio A, Broccoli V, Vicenzi E, Graham V, Pullan S, Dowall S, Hewson R, Jurt S, Zerbe O, Stettler K, Lanzavecchia A, Sallusto F, Cavalli A, Harris E, Lok SM, Varani L, Corti D. 2017. A human bi-specific antibody against Zika virus with high therapeutic potential. Cell 171:229–241. 10.1016/j.cell.2017.09.002. 28938115 PMC5673489

[52] Chen M, Puschmann TB, Marasek P, Inagaki M, Pekna M, Wilhelmsson U, Pekny M. 2018. Increased neuronal differentiation of neural progenitor cells derived from phosphovimentin-deficient mice. Mol Neurobiol 55:5478–5489. 10.1007/s12035-017-0759-0. 28956310 PMC5994207

[53] Ledur PF, Karmirian K, Pedrosa C, Souza LRQ, Assis-de-Lemos G, Martins TM, Ferreira J, de Azevedo Reis GF, Silva ES, Silva D, Salerno JA, Ornelas IM, Devalle S, Madeiro da Costa RF, Goto-Silva L, Higa LM, Melo A, Tanuri A, Chimelli L, Murata MM, Garcez PP, Filippi-Chiela EC, Galina A, Borges HL, Rehen SK. 2020. Zika virus infection leads to mitochondrial failure, oxidative stress and DNA damage in human iPSC-derived astrocytes. Sci Rep 10:1218. 10.1038/s41598-020-57914-x. 31988337 PMC6985105

[54] Rasmussen SA, Jamieson DJ, Honein MA, Petersen LR. 2016. Zika virus and birth defects–reviewing the evidence for causality. N Engl J Med 374:1981–1987. 10.1056/NEJMsr1604338. 27074377

[55] Turrini F, Ghezzi S, Pagani I, Poli G, Vicenzi E. 2016. Zika virus: a re-emerging pathogen with rapidly evolving public health implications. New Microbiol 39:86–90. 27196545

[56] Wen Z, Song H, Ming GL. 2017. How does Zika virus cause microcephaly? Genes Dev 31:849–861. 10.1101/gad.298216.117. 28566536 PMC5458753

[57] Li Y, Sun C, Yates EA, Jiang C, Wilkinson MC, Fernig DG. 2016. Heparin binding preference and structures in the fibroblast growth factor family parallel their evolutionary diversification. Open Biol 6:150275. 10.1098/rsob.150275. 27030175 PMC4821243

[58] Raballo R, Rhee J, Lyn-Cook R, Leckman JF, Schwartz ML, Vaccarino FM. 2000. Basic fibroblast growth factor (Fgf2) is necessary for cell proliferation and neurogenesis in the developing cerebral cortex. J Neurosci 20:5012–5023. 10.1523/jneurosci.20-13-05012.2000. 10864959 PMC6772267

[59] Schlessinger J, Plotnikov AN, Ibrahimi OA, Eliseenkova AV, Yeh BK, Yayon A, Linhardt RJ, Mohammadi M. 2000. Crystal structure of a ternary FGF-FGFR-heparin complex reveals a dual role for heparin in FGFR binding and dimerization. Mol Cell 6:743–750. 10.1016/s1097-2765(00)00073-3. 11030354

[60] Zhang L. 2010. Glycosaminoglycan (GAG) biosynthesis and GAG-binding proteins. Prog Mol Biol Transl Sci 93:1–17. 10.1016/S1877-1173(10)93001-9. 20807638

[61] Caldwell MA, Svendsen CN. 1998. Heparin, but not other proteoglycans potentiates the mitogenic effects of FGF-2 on mesencephalic precursor cells. Exp Neurol 152:1–10. 10.1006/exnr.1998.6815. 9682007

[62] Qian X, Song H, Ming GL. 2019. Brain organoids: advances, applications and challenges. Development 146:dev166074. 10.1242/dev.166074. 30992274 PMC6503989

[63] Clark AE, Zhu Z, Krach F, Rich JN, Yeo GW, Spector DH. 2021. Zika virus is transmitted in neural progenitor cells via cell-to-cell spread and infection is inhibited by the autophagy inducer trehalose. J Virol 95:e02024-20. 10.1128/JVI.02024-20. PMC8092816 33328307

[64] Kim SY, Zhao J, Liu X, Fraser K, Lin L, Zhang X, Zhang F, Dordick JS, Linhardt RJ. 2017. Interaction of Zika virus envelope protein with glycosaminoglycans. Biochemistry 56:1151–1162. 10.1021/acs.biochem.6b01056. 28151637 PMC7579681

[65] Skidmore MA, Kajaste-Rudnitski A, Wells NM, Guimond SE, Rudd TR, Yates EA, Vicenzi E. 2015. Inhibition of influenza H5N1 invasion by modified heparin derivatives. Med Chem Commun (Camb) 6:640–646. 10.1039/C4MD00516C.

[66] Lin Y-L, Lei H-Y, Lin Y-S, Yeh T-M, Chen S-H, Liu H-S. 2002. Heparin inhibits dengue-2 virus infection of five human liver cell lines. Antiviral Res 56:93–96. 10.1016/s0166-3542(02)00095-5. 12323403

[67] Walker SJ, Pizzato M, Takeuchi Y, Devereux S. 2002. Heparin binds to murine leukemia virus and inhibits Env-independent attachment and infection. J Virol 76:6909–6918. 10.1128/jvi.76.14.6909-6918.2002. 12072492 PMC136325

[68] Nahmias AJ, Kibrick S. 1964. Inhibitory effect of heparin on herpes simplex virus. J Bacteriol 87:1060–1066. 10.1128/jb.87.5.1060-1066.1964. 4289440 PMC277146

[69] Jones KS, Petrow-Sadowski C, Bertolette DC, Huang Y, Ruscetti FW. 2005. Heparan sulfate proteoglycans mediate attachment and entry of human T-cell leukemia virus type 1 virions into CD4^+^ T cells. J Virol 79:12692–12702. 10.1128/JVI.79.20.12692-12702.2005. 16188972 PMC1235841

[70] Kim SY, Koetzner CA, Payne AF, Nierode GJ, Yu Y, Wang R, Barr E, Dordick JS, Kramer LD, Zhang F, Linhardt RJ. 2019. Glycosaminoglycan compositional analysis of relevant tissues in Zika virus pathogenesis and in vitro evaluation of heparin as an antiviral against Zika virus infection. Biochemistry 58:1155–1166. 10.1021/acs.biochem.8b01267. 30698412 PMC7686953

[71] Olmo IG, Carvalho TG, Costa VV, Alves-Silva J, Ferrari CZ, Izidoro-Toledo TC, da Silva JF, Teixeira AL, Souza DG, Marques JT, Teixeira MM, Vieira LB, Ribeiro FM. 2017. Zika virus promotes neuronal cell death in a non-cell autonomous manner by triggering the release of neurotoxic factors. Front Immunol 8:1016. 10.3389/fimmu.2017.01016. 28878777 PMC5572413

[72] Ottoboni L, von Wunster B, Martino G. 2020. Therapeutic plasticity of neural stem cells. Front Neurol 11:148. 10.3389/fneur.2020.00148. 32265815 PMC7100551

[73] Greer I, Hunt BJ. 2005. Low molecular weight heparin in pregnancy: current issues. Br J Haematol 128:593–601. 10.1111/j.1365-2141.2004.05304.x. 15725079

[74] Taylor SL, Hogwood J, Guo W, Yates EA, Turnbull JE. 2019. By-products of heparin production provide a diverse source of heparin-like and heparan sulfate glycosaminoglycans. Sci Rep 9:2679. 10.1038/s41598-019-39093-6. 30804383 PMC6389988

[75] Hills FA, Abrahams VM, Gonzalez-Timon B, Francis J, Cloke B, Hinkson L, Rai R, Mor G, Regan L, Sullivan M, Lam EW, Brosens JJ. 2006. Heparin prevents programmed cell death in human trophoblast. Mol Hum Reprod 12:237–243. 10.1093/molehr/gal026. 16556679

[76] Kumar P, Mahajan S. 2013. Preimplantation and postimplantation therapy for the treatment of reproductive failure. J Hum Reprod Sci 6:88–92. 10.4103/0974-1208.117165. 24082648 PMC3778611

[77] Wan JG, Mu JS, Zhu HS, Geng JG. 2002. N-desulfated non-anticoagulant heparin inhibits leukocyte adhesion and transmigration in vitro and attenuates acute peritonitis and ischemia and reperfusion injury in vivo. Inflamm Res 51:435–443. 10.1007/pl00012403. 12365716

[78] Fritchley SJ, Kirby JA, Ali S. 2000. The antagonism of interferon-gamma (IFN-gamma) by heparin: examination of the blockade of class II MHC antigen and heat shock protein-70 expression. Clin Exp Immunol 120:247–252. 10.1046/j.1365-2249.2000.01178.x. 10792372 PMC1905634

[79] Spratte J, Schonborn M, Treder N, Bornkessel F, Zygmunt M, Fluhr H. 2015. Heparin modulates chemokines in human endometrial stromal cells by interaction with tumor necrosis factor alpha and thrombin. Fertil Steril 103:1363–1369. 10.1016/j.fertnstert.2015.02.023. 25813285

[80] Butti E, Cattaneo S, Bacigaluppi M, Cambiaghi M, Scotti G, Brambilla E, Sferruzza G, Ripamonti M, Simeoni F, Cacciaguerra L, Zanghì A, Quattrini A, Fesce R, Panina-Bordignon P, Giannese F, Cittaro D, Kuhlmann T, D’Adamo P, Rocca MA, Taverna S, Martino G. 2020. Neural precursor cells contribute to decision-making by tuning striatal connectivity via secretion of IGFBPL-1. bioRxiv. 10.1101/2020.12.29.424678.

[81] Reinhardt P, Glatza M, Hemmer K, Tsytsyura Y, Thiel CS, Hoing S, Moritz S, Parga JA, Wagner L, Bruder JM, Wu G, Schmid B, Ropke A, Klingauf J, Schwamborn JC, Gasser T, Scholer HR, Sterneckert J. 2013. Derivation and expansion using only small molecules of human neural progenitors for neurodegenerative disease modeling. PLoS One 8:e59252. 10.1371/journal.pone.0059252. 23533608 PMC3606479

